# Conformational study of L-methionine and L-cysteine derivatives through quantum chemical calculations and ^3^*J*_HH_ coupling constant analyses

**DOI:** 10.3762/bjoc.13.94

**Published:** 2017-05-17

**Authors:** Weslley G D P Silva, Carolyne B Braga, Roberto Rittner

**Affiliations:** 1 Chemistry Institute, University of Campinas, P.O. Box 6154, 13083−970, Campinas, SP, Brazil

**Keywords:** amino acid derivatives, conformational analysis, cysteine, methionine, NMR spectroscopy, quantum chemical calculations

## Abstract

The understanding of the conformational behavior of amino acids and their derivatives is a challenging task. Here, the conformational analysis of esterified and *N*-acetylated derivatives of L-methionine and L-cysteine using a combination of ^1^H NMR and electronic structure calculations is reported. The geometries and energies of the most stable conformers in isolated phase and taking into account the implicit solvent effects, according to the integral equation formalism polarizable continuum model (IEF−PCM), were obtained at the ωB97X-D/aug-cc-pVTZ level. The conformational preferences of the compounds in solution were also determined from experimental and theoretical ^3^*J*_HH_ coupling constants analysis in different aprotic solvents. The results showed that the conformational stability of the esterified derivatives is not very sensitive to solvent effects, whereas the conformational equilibrium of the *N*-acetylated derivatives changes in the presence of solvent. According to the natural bond orbital (NBO), quantum theory of atoms in molecules (QTAIM) and noncovalent interactions (NCI) methodologies, the conformational preferences for the compounds are not dictated by intramolecular hydrogen bonding, but by a joint contribution of hyperconjugative and steric effects.

## Introduction

Amino acids constitute the building blocks of proteins and peptides, which play an important role in numerous biological processes [1–2]. However, their studies in both isolated and condensed phases have been challenging for chemists and physicists due to the particular amino acid properties, such as high melting points, low vapor pressures and the occurrence of zwitterions in solution. Nevertheless, taking into account the recent experimental and theoretical developments, studies dealing with amino acids have been more widely reported, mainly in gas phase [3–8].

Among the 20 amino acids incorporated into proteins, L-methionine (L-Met) and L-cysteine (L-Cys) are the only two containing sulfur. The former is an initiator amino acid in the protein synthesis of all eukaryotics cells [9], whereas disulfide bonds formed by the oxidized thiol groups of cysteine confer exceptional stability for the peptides and proteins where they are present [10]. Thus, a systematic study on the conformational behavior of L-Met and L-Cys can reveal unique properties about the formation of proteins and peptides that happens in the biological environment.

The conformers of L-Met and L-Cys have been investigated by several experimental and theoretical methodologies, including FTIR [11], rotational and IR−UV double resonance spectroscopies [12–13], photon ionization mass spectrometry [14], X-ray absorption [15] and quantum chemical calculations [16–19]. In spite of these many studies performed, there is still a lack of information about the effects that rule their conformational isomerism. Additionally, the conformational flexibility of both amino acids leads to a variety of low energy geometries, which make their studies even more difficult.

An alternative capable of providing more detailed understanding about the structure and properties of more complex amino acids is the investigation of their esterified and *N*-acetylated derivatives. These derivatives are soluble in several organic solvents and thus, their properties can be studied through nuclear magnetic resonance (NMR), the most powerful spectroscopic characterization tool. For a deeper understanding of amino acid properties, an interplay between theoretical and experimental methods is crucial. Consequently, high-level quantum chemical calculations, such as the Møller–Plesset (MP2) method and density functional theory (DFT) calculations, together with experimental techniques have been combined to achieve more accurate results [20–24].

Some amino acid derivatives have been recently studied by our research group, including the derivatives of tryptophan [20], phenylalanine and tyrosine [21], aspartic acid [22], proline [23] and histidine [24]. These studies have provided significant results to understand the importance of the corresponding amino acids in processes in which they take part in the polypeptide chain. Furthermore, these works presented unique explanations about the conformational preferences of amino acids.

Therefore, it became of interest to extend the previous studies to investigate the conformational preferences of L-methionine and L-cysteine esterified and *N*-acetylated derivatives (Table 1). In order to obtain more insights about the main conformers and the operating effects in the compounds, both in isolated phase and in various aprotic solvents, ^1^H NMR spectroscopy and quantum chemical calculations, including natural bond orbitals (NBO), quantum theory of atoms in molecules (QTAIM), and noncovalent interactions (NCI) analyses were used.

**Table 1:** Depiction of the studied compounds (**1**–**4**).

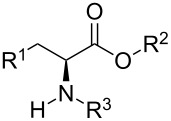			

compound	R^1^	R^2^	R^3^

L-methionine ethyl ester (**1**)	CH_2_SCH_3_	Et	H
L-cysteine methyl ester (**2**)	SH	Me	H
*N*-acetyl-L-methionine ethyl ester (**3**)	CH_2_SCH_3_	Et	COMe
*N*-acetyl-L-cysteine methyl ester (**4**)	SH	Me	COMe

## Results and Discussion

### Esterified derivatives of L-Met and L-Cys

**Figure 1:**
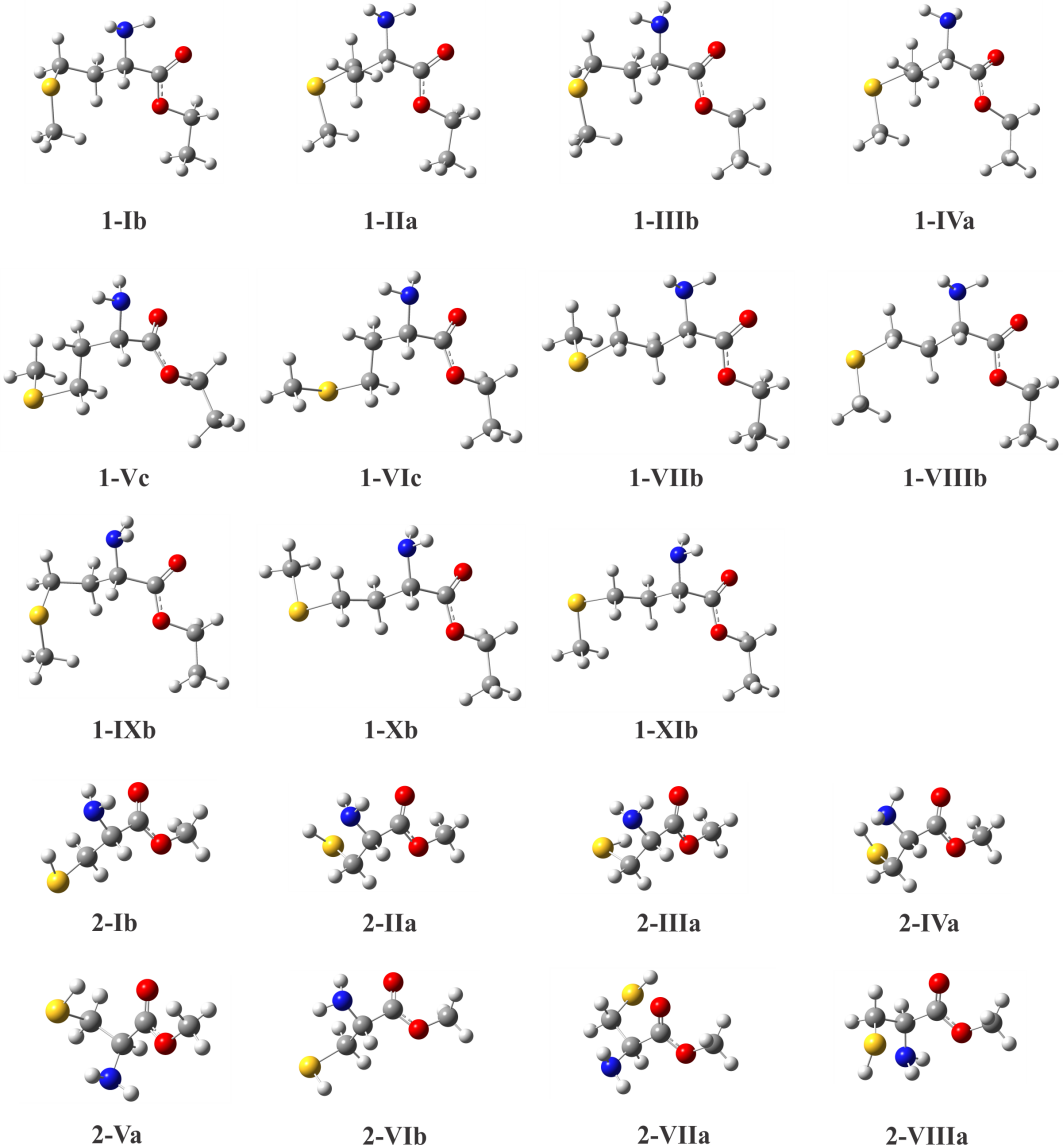
Most stable conformers of **1** and **2** obtained theoretically at the ωB97X-D/aug-cc-pVTZ level.

The lowest-energy conformers of **1** and **2** and their calculated parameters (ωB97X-D/aug-cc-pVTZ) in gas phase and in solution (IEF−PCM) are shown in the Figure 1 and Table 2, respectively.

**Table 2:** Calculated parameters (ωB97X-D/aug-cc-pVTZ) for the conformers of **1** and **2**. Relative Gibbs free energy (Δ*G*) and electronic energy with ZPE corrections (Δ*E*) are given in kcal mol^−1^, populations (*P*) in % and dihedral angles in degrees.

conformer	isolated		CHCl_3_		CH_2_Cl_2_		CH_3_CN		DMSO		dihedral angles	

	Δ*E*	*P*	Δ*E*	*P*	Δ*E*	*P*	Δ*E*	*P*	Δ*E*	*P*	H_a_−C−C−H_b1_	H_a_−C−C−H_b2_

**1-Ib**	–	–	0.00	24.6	0.00	19.8	0.05	17.0	0.06	16.8	−57.48	−174.32
**1-IIa**	1.38	5.2	0.23	16.6	0.08	17.2	0.05	17.0	0.06	16.9	64.78	−51.15
**1-IIIb**	1.97	2.0	0.37	13.2	0.14	15.7	0.00	18.5	0.00	18.6	−62.89	−178.89
**1-IVa**	1.99	1.9	0.46	11.3	0.28	12.3	0.33	10.6	0.33	10.8	64.89	−50.56
**1-Vc**	2.04	1.7	0.54	9.9	0.40	10.1	0.37	9.9	0.38	9.8	−179.14	65.24
**1-VIc**	2.27	1.2	0.59	9.1	0.37	10.6	0.31	11.0	0.32	10.9	−178.19	66.45
**1-VIIb**	–	–	0.65	8.2	0.55	7.9	0.45	8.6	0.46	8.5	−59.02	−176.61
**1-VIIIb**	–	–	0.74	7.1	0.67	6.4	0.54	7.4	0.52	7.7	−58.19	−175.66
**1-IXb**	0.00	53.9	–	–	–	–	–	–	–	–	−67.20	176.11
**1-Xb**	0.70	16.4	–	–	–	–	–	–	–	–	−68.81	174.67
**1-XIb**	0.66	17.7	–	–	–	–	–	–	–	–	−68.81	174.67

	Δ*G*	*P*	Δ*G*	*P*	Δ*G*	*P*	Δ*G*	*P*	Δ*G*	*P*	H_a_−C−C−H_b1_	H_a_−C−C−H_b2_

**2-Ib**	0.00	32.7	0.00	38.7	0.00	37.8	0.00	29.4	0.00	28.9	−64.21	176.88
**2-IIa**	0.21	23.0	0.50	16.6	0.52	15.8	0.33	16.9	0.32	16.9	59.63	−59.75
**2-IIIa**	0.30	19.6	0.44	18.5	0.44	18.0	0.24	19.7	0.23	19.7	65.19	−54.06
**2-IVa**	0.93	6.8	1.01	7.1	0.99	7.2	0.55	11.7	0.50	12.4	64.87	−114.09
**2-Va**	0.81	8.3	1.08	6.3	1.11	5.8	0.93	6.1	0.91	6.2	61.72	−57.86
**2-VIb**	1.31	3.6	1.30	4.3	1.16	5.3	0.91	6.3	0.91	6.2	−60.06	−179.39
**2-VIIa**	1.50	2.6	1.44	3.4	1.40	3.5	1.20	3.9	1.19	3.9	179.96	61.62
**2-VIIIa**	1.34	3.4	1.20	5.1	1.03	6.6	0.94	6.0	0.96	5.7	56.55	−63.06

**Figure 2:**
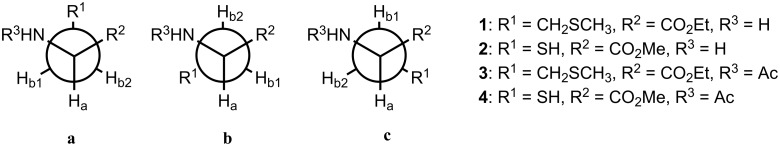
Three possible dispositions presented by geometries of the analyzed compounds **1**−**4**.

Each conformer of **1** and **2** (Figure 1) was named with a Roman numeral followed by a letter (**a**, **b** or **c**). The number represents the order of stability in chloroform for **1** and in isolated phase for **2**, while the letters denote the relationship between side and main chains, which are illustrated on the Newman projections of Figure 2. In the geometry **a**, hydrogen H_a_ is *gauche* to hydrogen atoms H_b1_ and H_b2_, while in the geometries **b** and **c**, H_a_ is *anti* to H_b2_ and H_b1_, respectively. These three possible dispositions were built based on the dihedral angles H_a_−C−C−H_b1_ and H_a_−C−C−H_b2_ depicted in Table 2.

The calculated populations (Table 2), derived from Δ*E* energies for **1** and Δ*G* for **2**, show that the most stable conformers of **1** and **2** are in the form **b** in both isolated phase and solution. Furthermore, the conformers **b** of the compound **1** are favored in the conformational equilibrium, since their populations together are larger than 50% of the total population of **1**, in all different studied media. Although the global minimum of **1** is in the form **b** in gas phase and also in solution, the three more stable geometries (**1-IXb**, **1-Xb** and **1-XIb**) in the former are substituted by three new structures (**1-Ib**, **1-VIIb** and **1-VIIIb**) when the solvent is introduced. Using the IEF−PCM implicit solvent model, small changes in the populations of conformers of **1** are observed when the solvent dielectric constant (ε) is increased. However, these slight variations do not affect the total populations of conformers **a**, **b** and **c**, and thus, it is an indicative that the conformers of **1** are not very sensitive to the solvent effect.

The same conformers were found in isolated phase and in solution for compound **2**. Moreover, its four more stable geometries (**2-Ib**, **2-IIa**, **2-IIIa** and **2-IVa**) represent approximately 80% of the conformational equilibrium (Table 2) in all different investigated media. As well as for **1**, geometries of **2** do not present significant variations when **ε** is increased. It also demonstrates that the solvent effect does not affect the conformer populations of **2**.

To obtain more details about the solvent effect in the conformational isomerism of the studied compounds, experimental NMR spectroscopy measurements and spin-spin coupling constant (^3^*J*_HH_) calculations were performed. The experimental ^1^H NMR data for **1** (Table 3) indicate that the ^3^*J*_HaHb1_ and ^3^*J*_HaHb2_ coupling constants are almost constant in the studied solvents, supporting our findings through theoretical calculations that the conformational equilibria of **1** are not affected by the solvent change. The two different observed values for ^3^*J*_HaHb1,obs_ (approximately 5.0 Hz) and ^3^*J*_HaHb2,obs_ (approximately 7.0 Hz) confirm that conformers in form **b** are favored in the equilibrium of **1**, since these constants are dependent on the dihedral angle H−C−C−H, according to the well-known Karplus relationship [25].

**Table 3:** Experimental and calculated ^1^H NMR data for the compound **1** in different solvents. The chemical shifts values are given in ppm and the ^3^*J*_HH_ coupling constants in Hz.

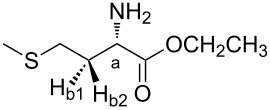								

solvent	ε	δH_a_	δH_b1_	δΗ_b2_	^3^*J*_HaHb1,obs_	^3^*J*_HaHb1,calc_	^3^*J*_HaHb2,obs_	^3^*J*_HaHb2,calc_

CDCl_3_	4.8	4.00	2.24	2.17	5.1 ± 0.05	4.6	7.1 ± 0.05	7.5
CD_2_Cl_2_	9.1	3.97	2.22	2.14	5.2 ± 0.05	4.8	7.0 ± 0.05	7.3
CD_3_CN	37.5	3.84	2.09	2.09	6.1 ± 0.05	4.7	6.1 ± 0.05	7.4
DMSO-*d*_6_	46.7	3.55	1.89	1.78	5.6 ± 0.05	4.7	7.4 ± 0.05	7.4

As the observed ^3^*J*_HH,obs_ coupling constant represents a weighted average of the contribution of each conformer, the calculated ^3^*J*_HH,calc_ spin−spin coupling constant represents the individual ^3^*J*_i_ coupling constant multiplied by the relative population (*n*_i_/*n*_j_) of each conformer i existent in the equilibrium, as shown by the Equation 1 [26]:

[1]
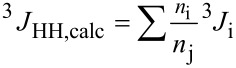


In this way, the averaged ^3^*J*_HaHb1,calc_ and ^3^*J*_HaHb2,calc_ (Table 3) obtained for the conformers of **1** are in good agreement with the experimental ones and reproduce well the results observed for this compound.

The study of **2** through NMR spectroscopy was not carried out in the present work because it was not possible to obtain the corresponding free amino acid derivative. When in solution, cysteine forms a dimer through disulfide bonds between the –SH groups and thus, it cannot be compared to the theoretically proposed compound. Another alternative would be the use of some chemical agent to break the disulfide bond in **2**, but it could induce changes in the conformational isomerism. However, the level of theory (ωB97X-D/aug-cc-pVTZ) used in this work for the studied compounds has shown good results for other amino acid derivatives, when comparing theoretical with experimental data [22–24]. Although **2** could not be experimentally studied, the theoretical calculations carried out strongly suggest that the conformational equilibrium of **2** and the populations of its conformers are not very sensitive to solvent effect.

When it comes to the study of amino acids and their derivatives, some studies found in the literature explain the conformational stability of amino acids solely by the formation of an intramolecular hydrogen bonding (IHB) [2,8,13]. Thus, to investigate the responsible effects governing the stability of the conformers of **1** and **2**, QTAIM, NCI and NBO methodologies were employed. These analyses were performed only for conformers that exist in solution.

In QTAIM analysis no bond path (BP) or bond critical point (BCP) are observed for the conformers of **1** and **2** between atoms where an IHB was expected, indicating no presence of IHB. Nevertheless, most conformers of **1** and **2** exhibit the NH_2_ group directed toward the oxygen atom of the carbonyl group and thus, an attractive interaction NH^…^O=C characteristic of an IHB would be expected. On the basis of some studies where QTAIM fails in describing the presence of weak long-range bonds [27–28], the NCI analysis was also performed. A comparison between the QTAIM and NCI analyses for conformers of **1** and **2** is presented in Figure S1 in Supporting Information File 1.

Although the QTAIM molecular graphs (Figure S1-a) do not display any BP or BCP related to an IHB for the studied geometries, the NCI analysis shows that most of the stable conformers present a NH^…^O=C-type attractive interaction, except the geometries **1-IIIb**, **2-IIa**, and **2-VIIa**. This interaction can be visualized through the NCI isosurfaces (Figure S1-b) and the plot of the reduced density gradient *S* versus sign (λ_2_)ρ(r) (Figure S1-c). Thus, the IHB NH^…^O=C found was characterized by trough with λ_2_ < 0 and the presence of a blue color in the sphere between the H(N) and O(C) atoms [29]. On the contrary, the non-observation of this interaction through QTAIM analysis is explained by the fact that troughs with λ_2_ < 0 do not reach *S* = 0, as described by Lane and co-workers [28].

The presence of the IHB NH^…^O=C was also investigated by the use of NBO analysis. The NBO calculations (Table 4) indicate that an IHB occurs in the conformers **1-Ib**, **1-VIIb**, **1-VIIIb** and **2-IVa**, because only these mentioned geometries present the hyperconjugative *n*_O_→σ*_N–H_ interaction. However, as these interactions are of small magnitude (0.72, 0.94, 0.86 and 1.32 kcal mol^−1^, respectively) and the other low-energy structures do not exhibit any significant *n*_O_→σ*_N–H_ interaction, it is possible to conclude that IHB is not the main governing effect of the conformational preferences of compounds **1** and **2**.

**Table 4:** Calculated NBO parameters (ωB97X-D/aug-cc-pVTZ) for the most stable conformers of the compounds **1** and **2**. Relative energy of the steric (*E*_rel,Lewis_) and hyperconjugative (*E*_rel,Hyper_) interactions are given in kcal mol^−1^. The sum of *E*_rel,Lewis_ and *E*_rel,Hyper_ is the total energy of the system.

conformer	CHCl_3_		DMSO		*n*_O_→σ*_N–H _^a^

	*E*_rel,Lewis_	*E*_rel,Hyper_	*E*_rel,Lewis_	*E*_rel,Hyper_	

**1-Ib**	0.89	1.66	1.76	2.27	0.72
**1-IIa**	1.84	2.44	1.92	2.51	−
**1-IIIb**	2.02	2.63	2.32	3.03	−
**1-IVa**	4.04	4.42	4.02	4.51	−
**1-Vc**	0.00	0.22	0.00	0.21	−
**1-VIc**	1.23	1.14	0.91	0.96	−
**1-VIIb**	0.18	0.00	0.25	0.00	0.94
**1-VIIIb**	0.54	0.38	0.38	0.31	0.86

**2-Ib**	0.00	0.00	1.74	2.76	−
**2-IIa**	2.05	2.11	4.76	5.98	−
**2-IIIa**	4.31	4.33	6.26	7.48	−
**2-IVa**	6.38	5.97	5.84	6.69	1.32
**2-Va**	3.59	2.95	7.62	8.03	−
**2-VIb**	2.14	1.26	0.00	0.00	−
**2-VIIa**	3.73	2.95	6.22	6.43	−
**2-VIIIa**	4.44	3.73	7.30	7.70	−

^a^NBO calculations were realized with an energy threshold of 0.5 kcal mol^−1^.

The contributions of steric (*E*_rel,Lewis_) and hyperconjugative (*E*_rel,Hyper_) effects (Table 4) indicate that the most destabilized conformers by steric hindrance are also the most stabilized ones by hyperconjugation, such as the conformers **1-Ib** and **2-Va**. In this way, the NBO investigation shows that not only is a specific interaction the responsible for the observed conformational preferences for the compounds **1** and **2**, but an interplay between hyperconjugation and steric hindrance.

### *N*-Acetylated derivatives of L-Met and L-Cys

**Figure 3:**
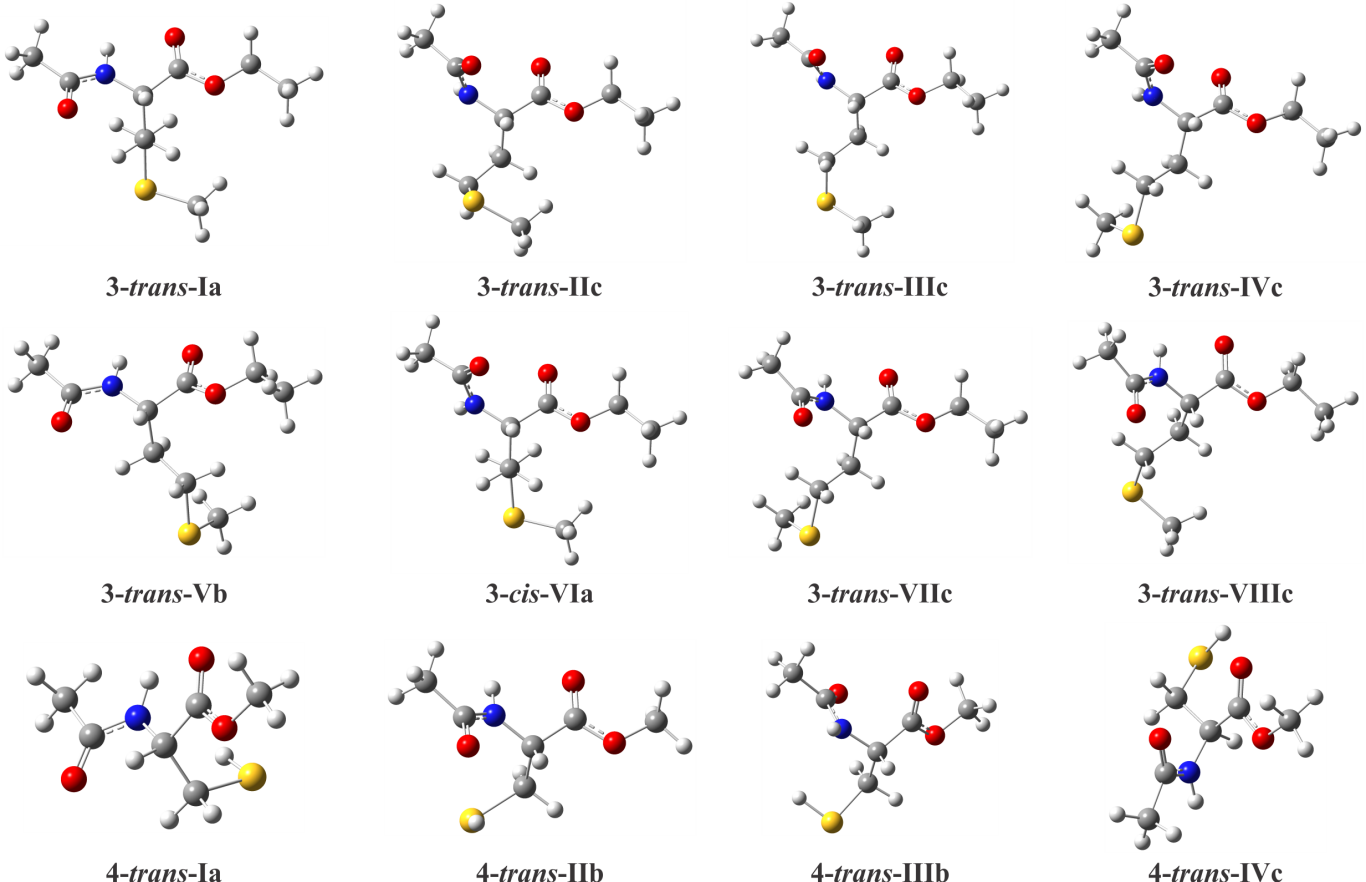
Most stable conformers of **3** and **4** obtained theoretically at the ωB97X-D/aug-cc-pVTZ level.

A similar study was performed for the derivatives **3** and **4**. The most stable conformers of **3** and **4** and their calculated parameters (ωB97X−D/aug−cc−pVTZ) in isolated phase and in solution are shown in Figure 3 and Table 5, respectively.

**Table 5:** Calculated parameters (ωB97X-D/aug-cc-pVTZ) for conformers of **3** and **4** in isolated phase and in solution (IEF−PCM). Relative Gibbs free energies (Δ*G*) and electronic energies with ZPE corrections (Δ*E*) are given in kcal mol^−1^, populations (*P*) in %, and dihedral angles in degrees.

conformer	isolated		CHCl_3_		CH_2_Cl_2_		CH_3_CN		DMSO		dihedral angles	

	Δ*E*	*P*	Δ*E*	*P*	Δ*E*	*P*	Δ*E*	*P*	Δ*E*	*P*	H_a_−C−C−H_b1_	H_a_−C−C−H_b2_

**3-*trans*-Ia**	0.00	45.6	0.00	54.9	0.00	45.7	0.00	36.4	0.00	34.8	62.03	−53.73
**3-*trans*-IIc**	1.44	4.0	0.60	19.9	0.51	19.4	0.16	27.6	0.13	27.8	174.83	−69.51
**3-*trans*-IIIc**	–	–	1.11	8.4	0.84	11.0	0.68	11.6	0.66	11.3	175.55	−67.86
**3-*trans*-IVc**	–	–	1.24	6.7	0.99	8.5	0.73	10.5	0.60	12.7	177.18	−66.41
**3-*trans*-Vb**	1.07	7.4	1.00	10.1	1.00	8.6	1.00	6.8	1.00	6.5	69.28	184.60
**3-*cis*-VIa**	–	–	–	–	1.13	6.8	0.98	7.1	0.96	6.9	62.43	−53.08
**3-*trans*-VIIc**	0.41	22.9	–	–	–	–	–	–	–	–	179.17	−64.16
**3-*trans*-VIIIc**	0.49	20.1	–	–	–	–	–	–	–	–	-177.35	−60.85

	Δ*G*	*P*	Δ*G*	*P*	Δ*G*	*P*	Δ*G*	*P*	Δ*G*	*P*	H_a_−C−C−H_b1_	H_a_−C−C−H_b2_

**4*-trans*-Ia**	0.00	97.2	0.00	87.0	0.00	82.0	0.00	69.7	0.00	67.7	62.60	−55.56
**4-*trans*-IIb**	2.17	2.5	1.21	11.2	0.98	15.7	0.57	26.8	0.51	28.7	−56.64	−175.04
**4*-trans*-IIIb**	3.79	0.2	2.68	0.9	2.68	0.9	2.47	1.1	2.47	1.1	−62.18	178.85
**4*-trans*-IVc**	3.87	0.1	2.72	0.9	2.42	1.4	1.98	2.4	1.93	2.5	−164.09	77.48

Each conformer of **3** and **4** was named with the *cis*−*trans* designation, which indicates the position of the amide linkage with respect to the C(O)OEt group in **3** and C(O)OMe in **4**, followed by a Roman numeral and a letter. The number represents the order of stability in chloroform, and the letter denotes the relationship between side and main chains (Figure 2). The presence of a larger number of dihedral angles in the compound **3** implies the existence of more stable conformers than **4**.

The calculated parameters (Table 5) indicate that the most stable conformers of **3** (**3-*trans*-Ia**) and **4** (**4*-trans*-Ia**) are *trans* in all different studied media. In isolated phase, the conformer **3-*trans*-Ia** represents 45.6% of the conformational equilibrium of **3**, but when the dielectric constant of the medium is increased, this geometry is destabilized and has its population decreased to 34.8%. The same tendency is observed for **4*-trans*-Ia,** which has its population reduced from almost 100% in isolated phase to 67.7% in DMSO. In general, these population changes for the geometries of compounds **3** and **4** indicate that their conformational equilibria are sensitive to solvent effects.

The conformational changes induced by the solvent were also investigated through experimental ^3^*J*_HH_ coupling constant obtained from ^1^H NMR spectroscopy and the corresponding calculated ones (Table 6). For the compound **3**, the experimental data show that the ^3^*J*_HaHb1,obs_ and ^3^*J*_HaHb2,obs_ coupling constants vary when the dielectric constant of the solvent (ε) is increased, corroborating the theoretical findings that the populations of its conformers are sensitive to the solvent effects. In chloroform, the difference between ^3^*J*_HaHb1,obs_ and ^3^*J*_HaHb2,obs_ (about 2.0 Hz) indicates that conformers **a** are favored in this solvent, as expected (54.9% from Table 5). When ε is increased, the difference between ^3^*J*_HaHb1,obs_ and ^3^*J*_HaHb2,obs_ increases to almost 4.0 Hz in DMSO and this fact is related to the stabilization of conformers **c**, which have H_a_* anti* to H_b1_, and *gauche* to H_b2_ (Table 5). Unlike **3**, the ^3^*J*_HaHb1,obs_ and ^3^*J*_HaHb2,obs_ for compound **4** exhibit close values in CHCl_3_ (3.9 and 4.3 Hz, respectively), and this also suggests the predominance of conformers **a** in this less polar solvent. However, despite conformers **a** of **4** being more populated in all studied media, ^3^*J*_HaHb2,obs_ increases from 4.3 Hz in CDCl_3_ to 7.5 Hz in DMSO, and it indicates the stabilization of conformers **b** in more polar solvents, which have H_a_ *anti* to H_b2_. Overall, the averaged ^3^*J*_HH,calc_ coupling constants obtained for the conformers of **3** and **4** (Table 6) are in a good agreement with the experimental data and both reproduce well the observed trend for these compounds.

**Table 6:** Experimental and calculated ^1^H NMR data for the compounds **3** and **4** in different solvents. The chemical shifts values are given in ppm and the ^3^*J*_HH_ coupling constants in Hz.

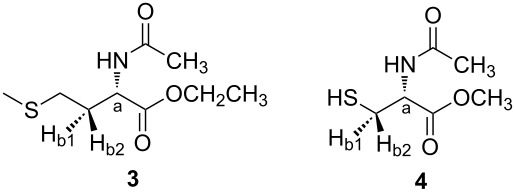											

compound	solvent	ε	δΗ(N)	δH_a_	δH_b1_	δΗ_b2_	^3^*J*_HaHb1,obs_	^3^*J*_HaHb1,calc_	^3^*J*_HaHb2,obs_	^3^*J*_HaHb2,calc_	^3^*J*_HaH(N)_

**3**	CDCl_3_	4.8	6.18	4.70	2.17	1.98	7.1 ± 0.07	7.0	5.1 ± 0.07	4.0	7.0 ± 0.07
	CD_2_Cl_2_	9.1	6.18	4.62	2.13	1.95	7.7 ± 0.07	7.3	5.1 ± 0.07	3.9	5.5 ± 0.07
	CD_3_CN	37.5	6.70	4.45	2.03	1.89	8.6 ± 0.07	8.1	4.9 ± 0.07	3.7	–
	DMSO-*d*_6_	46.7	8.26	4.31	1.92	1.84	9.1 ± 0.07	8.2	4.9 ± 0.07	3.7	7.4 ± 0.07

**4**	CDCl_3_	4.8	6.43	4.90	3.03	3.00	3.9 ± 0.05	3.1	4.3 ± 0.05	4.1	7.8 ± 0.05
	CD_2_Cl_2_	9.1	6.47	4.83	3.00	2.98	4.3 ± 0.05	3.2	4.4 ± 0.05	4.4	7.5 ± 0.05
	CD_3_CN	37.5	6.84	4.62	2.91	2.86	4.8 ± 0.05	3.5	6.0 ± 0.05	6.3	7.9 ± 0.05
	DMSO-*d*_6_	46.7	8.32	4.44	2.83	2.74	5.0 ± 0.05	3.9	7.5 ± 0.05	6.6	7.6 ± 0.05

**Figure 4:**
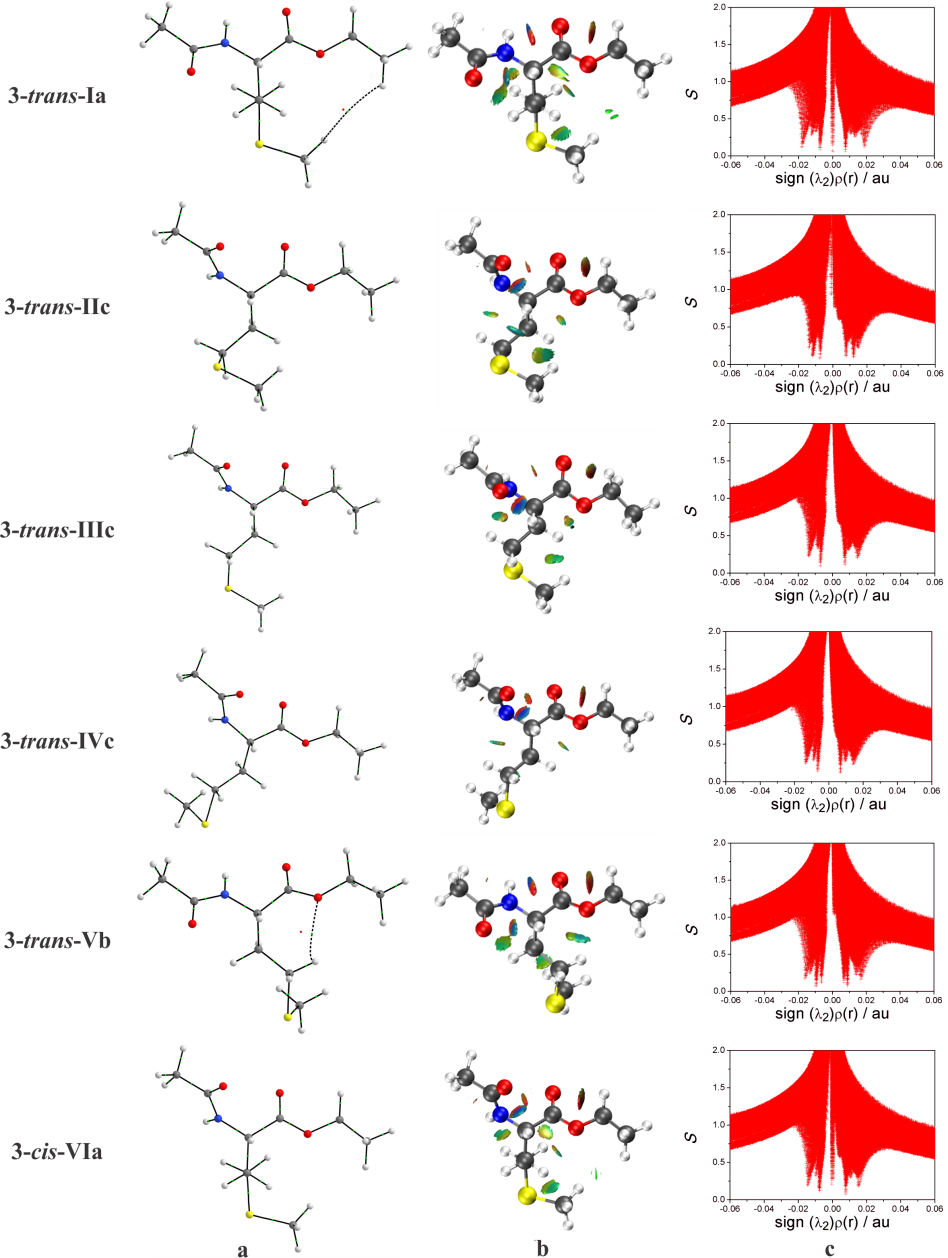
(a) QTAIM molecular graphs [30]; (b) NCI isosurfaces generated with *S* = 0.6 au and blue−green−red scaling from −0.02 < (λ_2_)ρ(r) < 0.02 au, and (c) NCI plots of the reduced density gradients *S* versus sign (λ_2_)ρ(r) for the conformers of **3**.

**Figure 5:**
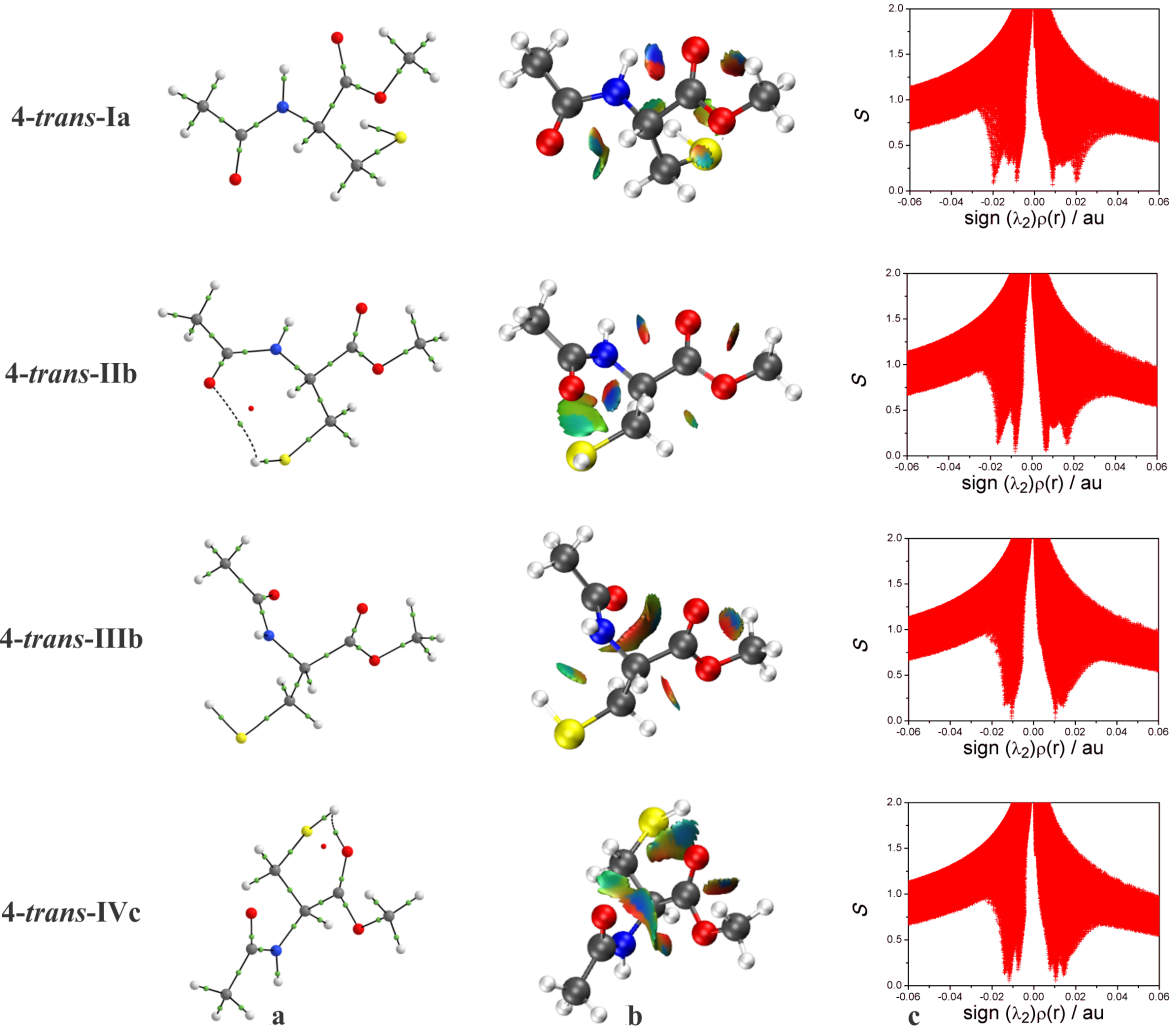
(a) QTAIM molecular graphs; (b) NCI isosurfaces generated with *S* = 0.6 au and blue−green−red scaling from −0.02 < (λ_2_)ρ(r) < 0.02 au, and (c) NCI plots of the reduced density gradients *S* versus sign (λ_2_)ρ(r) for the conformers of **4**.

Aiming to explain the higher stabilities showed by the conformers **3-*trans*-Ia** and **4-*trans*-Ia** in all studied media, QTAIM, NCI, and NBO approaches were carried out for the conformers of **3** and **4** existing in solution (Figure 4 and Figure 5). For example, in QTAIM analysis for the conformers of **4** (Figure 5a), a BP and a BCP regarding the IHB were observed only for **4-*trans*-IIb** and **4-*trans*-IVc**, demonstrating the presence of a S−H^…^O−like IHB and, consequently, the formation of a six and seven−membered ring, respectively. In agreement with the QTAIM, NCI methodology (Figure 5b and Figure 5c) confirms the presence of this S−H^…^O interaction in the conformers **4-*trans*-IIb** and **4-*trans*-IVc** (λ_2_ < 0 in the NCI plot, and a blue color in the sphere between the carbonyl oxygen and the H(S) atom in the NCI isosurface). In addition, NCI analysis also indicates the presence of an IHB NH^…^O=C (not observed in QTAIM) in conformers **3-*trans*-Ia**, **3-*trans*-Vb**, **4-*trans*-Ia**, and **4-*trans*-IIb**.

In order to evaluate also the presence of IHB, as well as the influences of steric and hyperconjugative interactions on the conformational isomerism of **3** and **4**, NBO analysis was employed. The NBO calculations (Table 7) confirm the presence of IHB in conformers **3-*trans*-Ia**, **3-*trans*-Vb**, and **4-*trans*-Ia**, evidenced by the *n*_O_→σ*_N–H_ and *n*_O_→σ*_S–H_ hyperconjugative interactions. Similarly to the results obtained for the compounds **1** and **2**, these interactions are of small magnitude (Table 7), and their presence is not related to the order of energy observed for the studied conformers, indicating that the IHB does not play a major role in the stability of the conformers of **3** and **4**. Also analogously to **1** and **2**, the conformational preferences observed for the conformers of the *N*-acetylated derivatives result from an interplay between steric repulsion and hyperconjugation.

**Table 7:** NBO parameters for conformers of compounds **3** and **4**, calculated at the ωB97X-D/aug-cc-pVTZ level of theory. Relative energy of the steric (*E*_rel,Lewis_) and hyperconjugative (*E*_rel,Hyper_) interactions are given in kcal mol^−1^. The sum of *E*_rel,Lewis_ and *E*_rel,Hyper_ is the total energy of the system.

conformation	CHCl_3_		DMSO		*n*_O_→σ*_N–H _^a^	*n*_O_→σ*_S–H _^a^

	*E*_rel,Lewis_	*E*_rel,Hyper_	*E*_rel,Lewis_	*E*_rel,Hyper_		

**3-*trans*-Ia**	7.47	8.65	5.19	5.90	0.98	–
**3-*trans*-IIc**	3.13	3.63	3.11	3.91	–	–
**3-*trans*-IIIc**	1.17	1.44	0.16	0.35	–	–
**3-*trans*-IVc**	0.00	0.00	0.00	0.00	–	–
**3-*trans*-Vb**	3.85	3.75	0.92	0.39	0.70	–
**3-*cis*-VIa**	–	–	4.63	4.12	–	–

**4*-trans*-Ia**	2.32	2.72	3.14	2.54	1.03	–
**4-*trans*-IIb**	0.00	0.00	0.00	0.00	–	–
**4*-trans*-IIIb**	3.86	1.63	5.94	2.51	–	–
**4*-trans*-IVc**	5.26	3.24	7.15	4.99	–	0.86

^a^NBO calculations were realized with an energy threshold of 0.5 kcal mol^−1^.

## Conclusion

In summary, the use of quantum chemical calculations and ^3^*J*_HH_ coupling constant analyses, in the present work, allowed the determination of the conformational preferences of methionine and cysteine esterified and *N*−acetylated derivatives in isolated phase and in different aprotic solvents. A comparison between calculated and experimental ^3^*J*_HH_ coupling constants indicated that the conformational isomerism of compounds **1** and **2** is not very sensitive to solvent effects. On the other hand, the conformers of **3** and **4** had their populations changed when the solvent effects were taken into account.

NBO, QTAIM and NCI methodologies showed that the presence of a NH^…^O=C−like IHB in some of the studied conformers is not related to their stabilities and, thus, more than just a specific interaction is governing the conformational isomerism of the compounds **1**, **2**, **3**, and **4**. The observed conformational preferences for these derivatives are due to a combination of steric hindrance and hyperconjugative effects.

To sum up, the obtained results in the present study are a good illustration of the nature of amino acids derivatives in solution. Furthermore, these results can be extended to the understanding of the conformational behavior of methionine and cysteine amino acid in the biological environment, such as in polypeptide chains.

## Experimental

### Synthesis of compounds **1**, **3** and **4**

Compound **1** was purchased from Sigma-Aldrich in the form of chloridrate and deprotonated using activated zinc dust, as described in the literature for similar compounds [31]. Compounds **3** and **4** were obtained by the esterification of the corresponding *N*-acetyl-L-amino acids (Sigma-Aldrich), following a known procedure [23,32]. The detailed syntheses are described in Supporting Information File 1.

### Spectroscopic measurements

^1^H NMR spectra for **1**, **3** and **4** were recorded on a Bruker Avance III operating at 600.17 MHz for hydrogen nuclei. Compound **2** was not experimentally studied, since it dimerized during the measurements to give the corresponding disulfide derivative. Spectra were acquired using solutions of ca. 10 mg of solute in 0.7 mL of deuterated solvents (CDCl_3_, CD_2_Cl_2_, CD_3_CN and DMSO-*d*_6_), referenced to internal TMS. Typical acquisition and processing conditions are shown in the NMR spectra provided in Supporting Information File 1 (Figures S2–S13).

### Computational details

**Figure 6:**
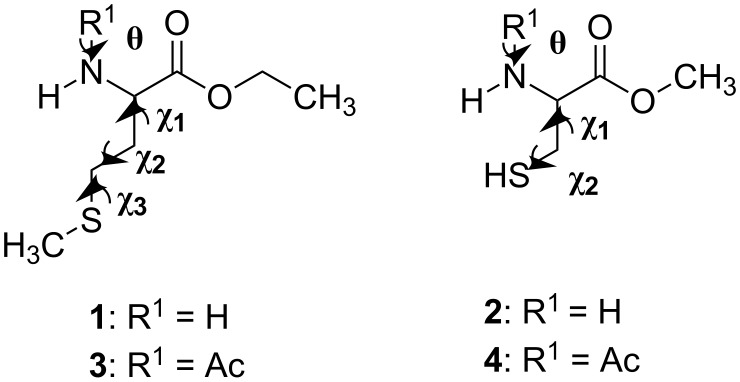
Definition of the selected dihedral angles for the studied compounds.

The starting conformer geometries for **1** and **2** were constructed from the six most stable optimized conformers of L-alanine methyl ester (Ala-OMe), reported by a previous study [33], which have the less energetic arrangement of the backbone [CH_3_−O−C(O)−CH(NH_2_)−], as follows. A methyl hydrogen atom (side chain) of Ala-OMe was substituted by CH_2_−S−CH_3_ and S−H, giving rise to six new geometries for the compounds **1** and **2**, respectively. Moreover, for **1**, at the beginning of the backbone a methyl group was added to the Ala-OMe structures by replacing a hydrogen atom of their methyl groups. Thus, six potential energy curves (PEC’s) for **1** and six potential surfaces (PES, Figure S14 of Supporting Information File 1) for **2** were built from these six new structures of the compounds by scanning all torsional angles of the side chain (Figure 6) in 36 steps of 10° each, from 0° to 360°, at the B3LYP/cc-pVDZ level. In this step, the dihedral angles of the backbone were kept fixed. 38 and 34 different energy minima were identified for **1** and **2**, respectively.

The 34 geometries of **2** were fully reoptimized without restrictions using the B3LYP [34–35], B3LYP-D3 [36], CAM-B3LYP [37], M05-2X [38], M06-2X [39], B97-D [40] and ωB97X-D [41] functionals, and the ab initio MP2 method [42], with the aug-cc-pVTZ basis set [43], in order to achieve accurate energy and geometry results and at a reasonable computational cost. Since the ωB97X-D/aug-cc-pVTZ level presented one of the smallest mean absolute deviation from MP2/aug-cc-pVTZ single point calculations (Table S1, Supporting Information File 1), assumed as the reference level, and as the ωB97X-D DFT functional has been recognized to reliably treat noncovalent interactions as well as to present good proximity with spectroscopic results [22–24], it was used in all subsequent calculations. These calculations were only performed for the conformers of **2** due to their size compared to the geometries of the L-Met esterified derivative. Then, beyond the conformers of the compound **2**, the conformers of **1** were also fully optimized at the ωB97X-D/aug-cc-pVTZ level in both isolated phase and implicit solvent (chloroform, dichloromethane, acetonitrile, and dimethyl sulfoxide), according to the Integral Equation Formalism Polarizable Continuum Model (IEF−PCM) [44]. As expected, the ωB97X-D/aug-cc-pVTZ theoretical level showed good performance for these derivatives in comparison to similar systems previously studied [12,17,19], where higher levels of theory were used. These optimization calculations resulted in 11 stable conformers for **1** and in 8 for **2**, which were taken into account in the discussion of the results. The other geometries, with relative energies over 2.0 kcal mol^−1^, were discarded because they do not contribute to the conformational equilibrium of the studied compounds. Frequency calculations with ZPE corrections were carried out to guarantee the absence of imaginary frequencies in the geometries. Spin-spin coupling constants (^3^*J*_HH_) were calculated for each conformer in the IEF−PCM model using the ωB97X-D functional and EPR-III (for C and H atoms) [45] and aug-cc-pVTZ [43] (for O, N and S atoms) basis sets. All calculations cited above were performed using the Gaussian 09 program [46].

Possible intramolecular interactions were evaluated through natural bond orbital (NBO) analysis, at ωB97X-D/aug-cc-pVTZ level, using NBO 5.9 program [47]. In addition, quantum theory of atoms in molecules (QTAIM) [48] and noncovalent interactions (NCI) [49] analyses were carried out for the same purpose using the AIMALL [50] and NCIPLOT [29] programs, respectively.

Similar calculations were performed for the corresponding *N*-acetylated derivatives, **3** and **4**, using the same previously employed level of theory. The geometries for the conformers of **3** and **4** were constructed from the 11 and 8 most stable geometries obtained for **1** and **2**, respectively, by replacing one hydrogen atom of the amine group by the C(O)Me group, resulting in an amide linkage. Each structure of the *N*-acetylated derivatives presented two possible stereoisomers, i.e., where the dihedral angle θ [C−N−C(O)−C] (Figure 6) can be both 0° and 180°. Thus, the resulting 22 and 16 possible geometries of **3** and **4**, respectively, were optimized. The optimization calculations gave rise to 8 and 4 stable conformers for **3** and **4**, respectively.

## Supporting Information

File 1 QTAIM and NCI molecular graphs for the most stable conformers of compounds **1** and **2**; ^1^H NMR spectra for the studied compounds; potential energy surfaces and contour maps for the L-cysteine methyl ester; comparison of the energies, populations and other relevant structural parameters for the conformers of the L-cysteine methyl ester in several theoretical levels; detailed procedures for preparation of the compounds.
