# Supplementary material for: Conformational study of L-methionine and L-cysteine derivatives through quantum chemical calculations and 3JHH coupling constant analyses
Source: Beilstein J Org Chem. 2017 May 17;13:925–37. doi: 10.3762/bjoc.13.94 (PMC5480334; doi:10.3762/bjoc.13.94)
Supplement: File 1 — QTAIM and NCI molecular graphs for the most stable conformers of compounds 1 and 2; 1H NMR spectra for the studied compounds; potential energy surfaces and contour maps for the L-cysteine methyl ester; comparison of the energies, populations and other relevant structural parameters for the conformers of the L-cysteine methyl ester in several theoretical levels; detailed procedures for preparation of the compounds. [file Beilstein_J_Org_Chem-13-925-s001.pdf]

Supporting Information

for

Conformational study of L-methionine and L-cysteine derivatives through quantum chemical calculations and $^{3}J_{HH}$ coupling constant analyses

Weslley G. D. P. Silva,[‡] Carolyne B. Braga[*‡] and Roberto Rittner

Chemistry Institute, University of Campinas, P.O. Box 6154, 13083−970, Campinas, SP, Brazil

E-mail: Carolyne B. Braga - carolyne.braga@iqm.unicamp.br

*Corresponding author

‡Authors contributed equally to this work

QTAIM and NCI molecular graphs for the most stable conformers of compounds 1 and 2; ^{1}H NMR spectra for the studied compounds; potential energy surfaces and contour maps for the L-cysteine methyl ester; comparison of the energies, populations and other relevant structural parameters for the conformers of the L-cysteine methyl ester in several theoretical levels; detailed procedures for preparation of the compounds.

1-Ib

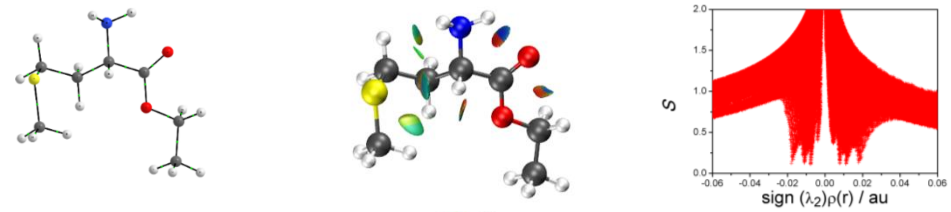

1-IIa

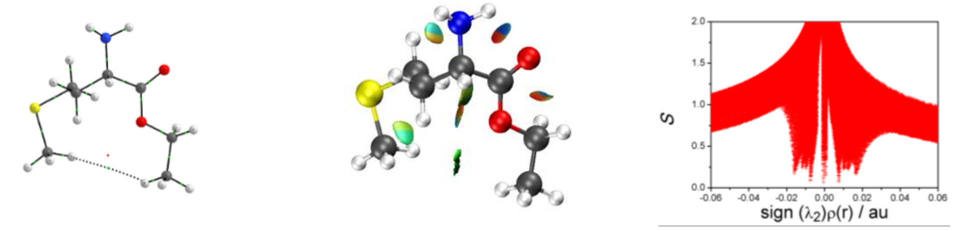

1-IIIb

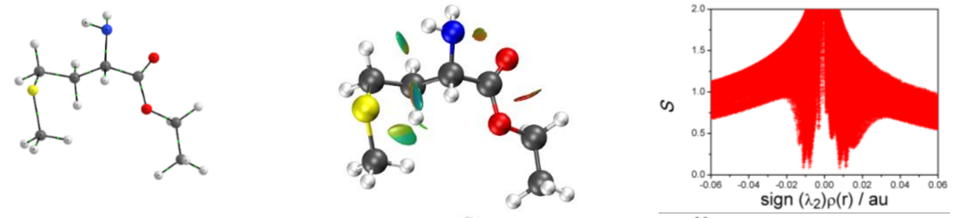

1-IVa

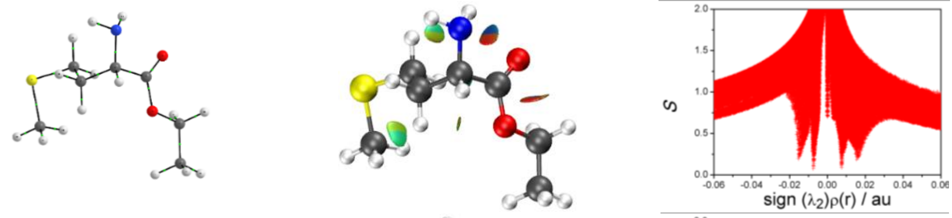

1-Vc

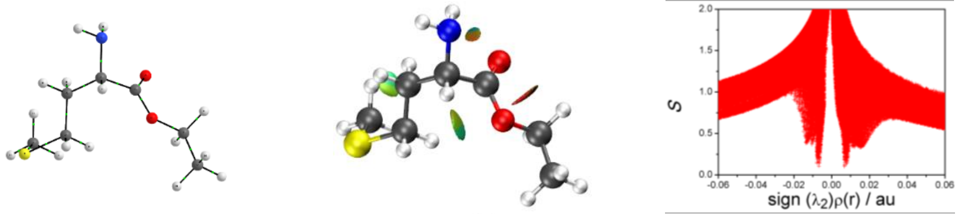

1-VIc

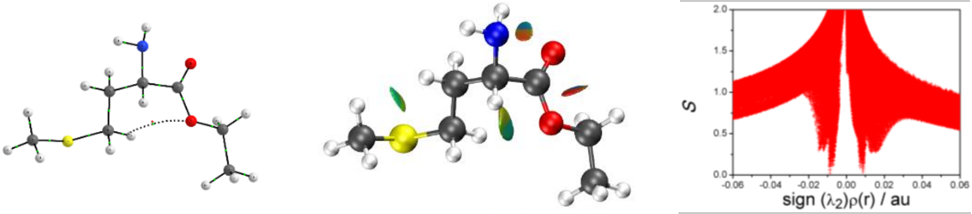

1-VIIb

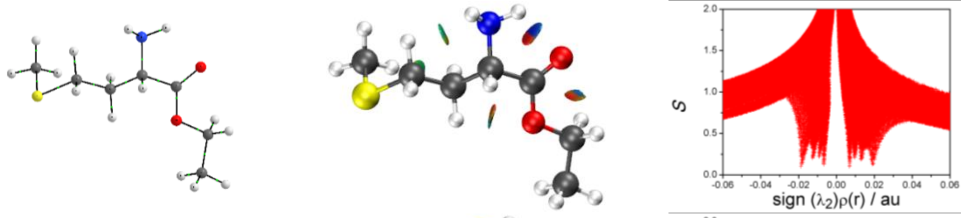

1-VIIIb

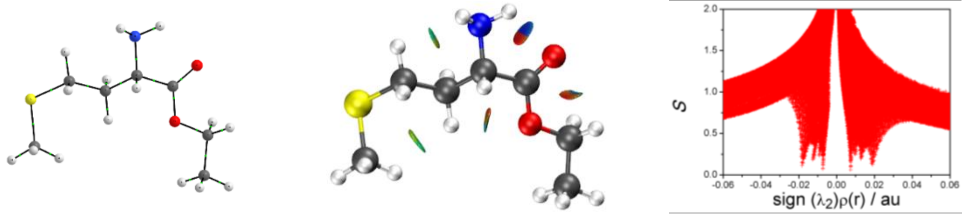

(to be continued)

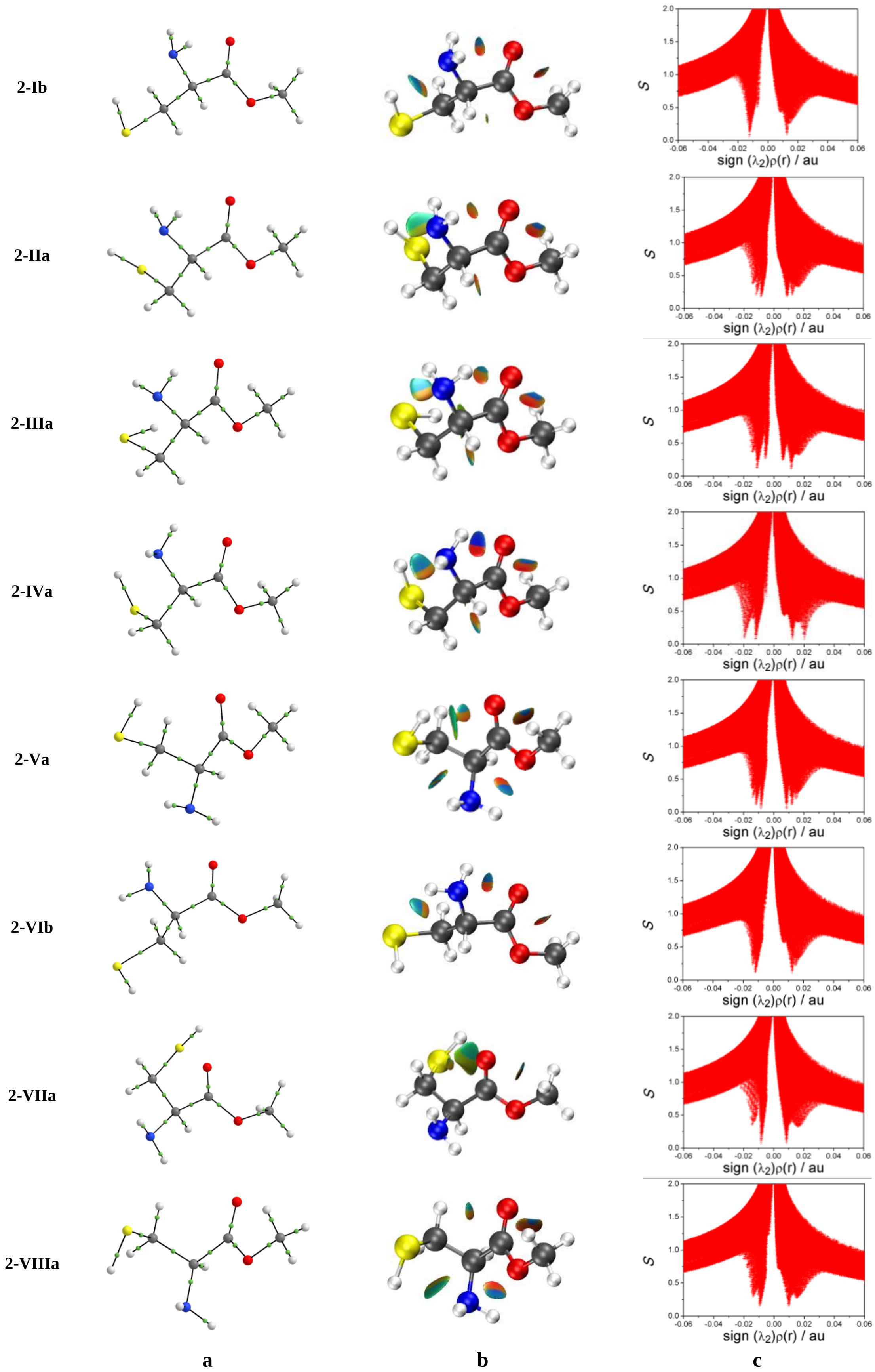

Figure S1: (a) QTAIM molecular graphs; (b) NCI isosurfaces generated with s = 0.5 au and blue−green−red scaling from -0.02 < $(\lambda_2)\rho(r)$ < 0.02 au, and (c) NCI plots of the reduced density gradients S versus sign $(\lambda_2)\rho(r)$ for the conformers of **1** and **2**.

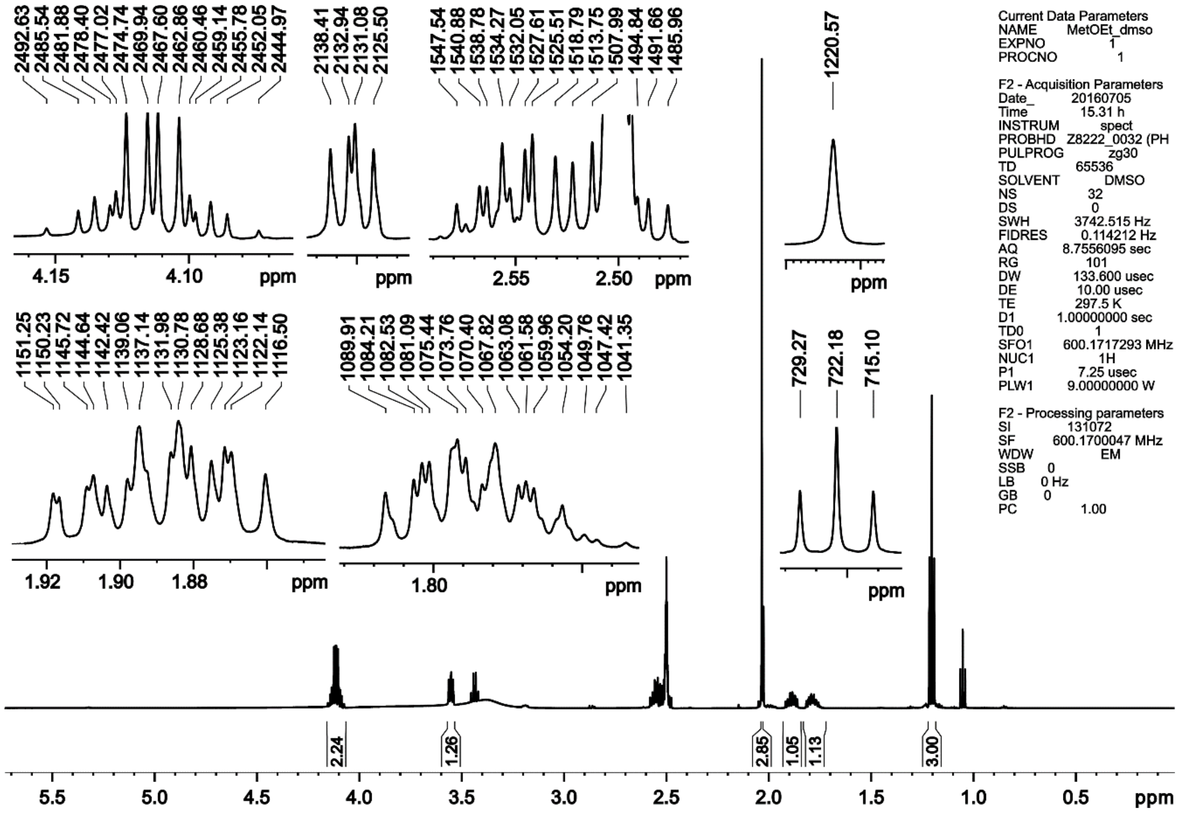

Figure S2. Compound **1** ^{1}H NMR spectrum in DMSO-d_6 at 25 °C.

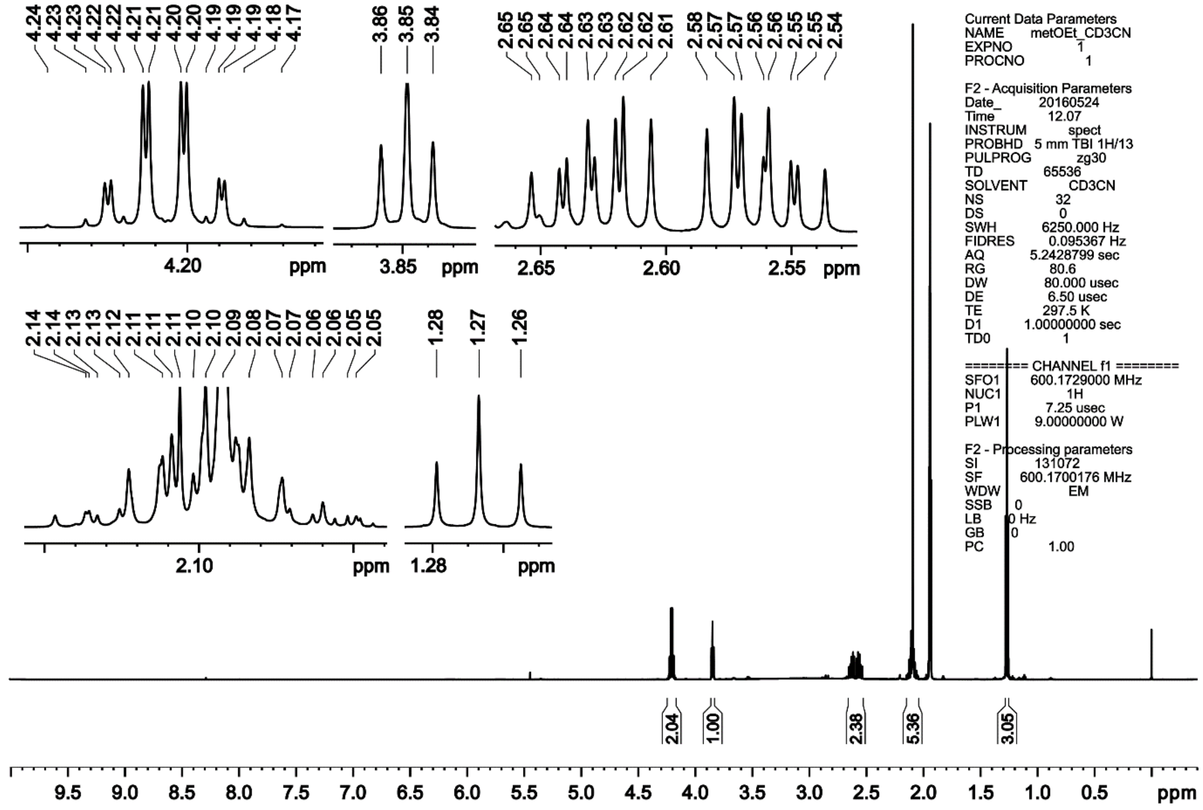

Figure S3: Compound **1** ^{1}H NMR spectrum in CD_3CN at 25 °C.

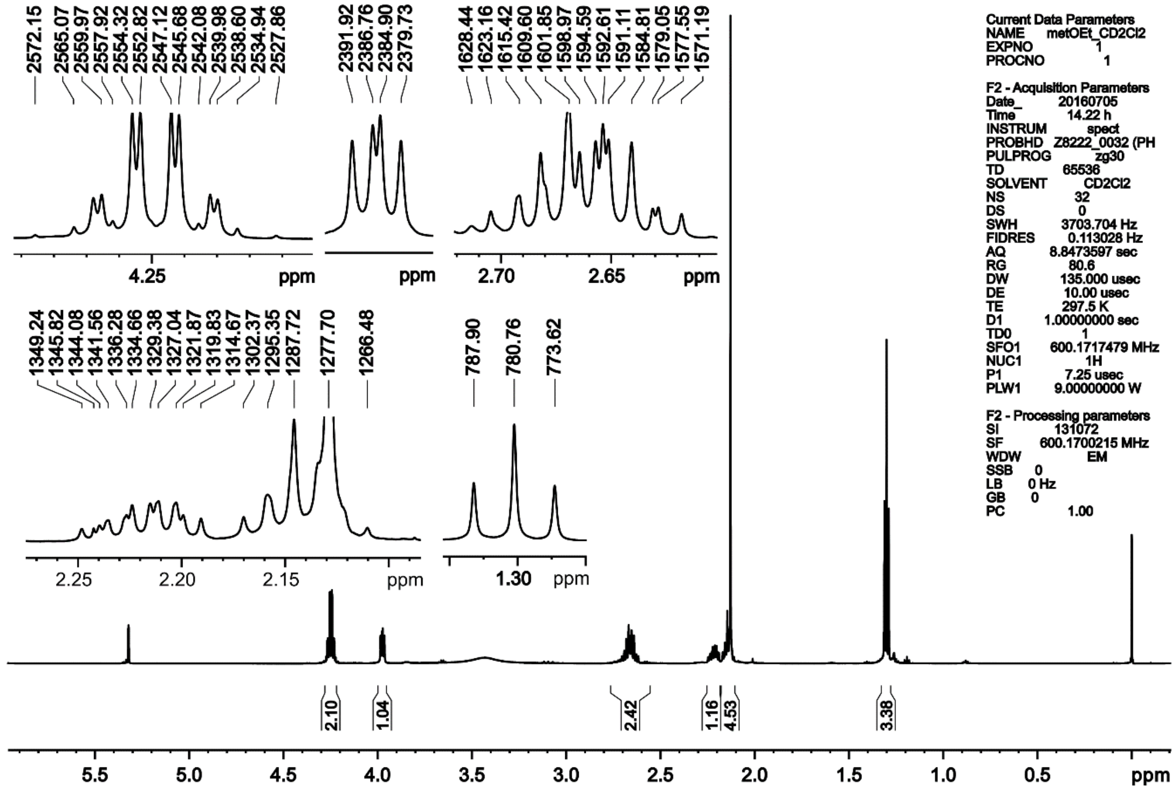

Figure S4: Compound **1** ^{1}H NMR spectrum in CD_2Cl_2 at 25 °C.

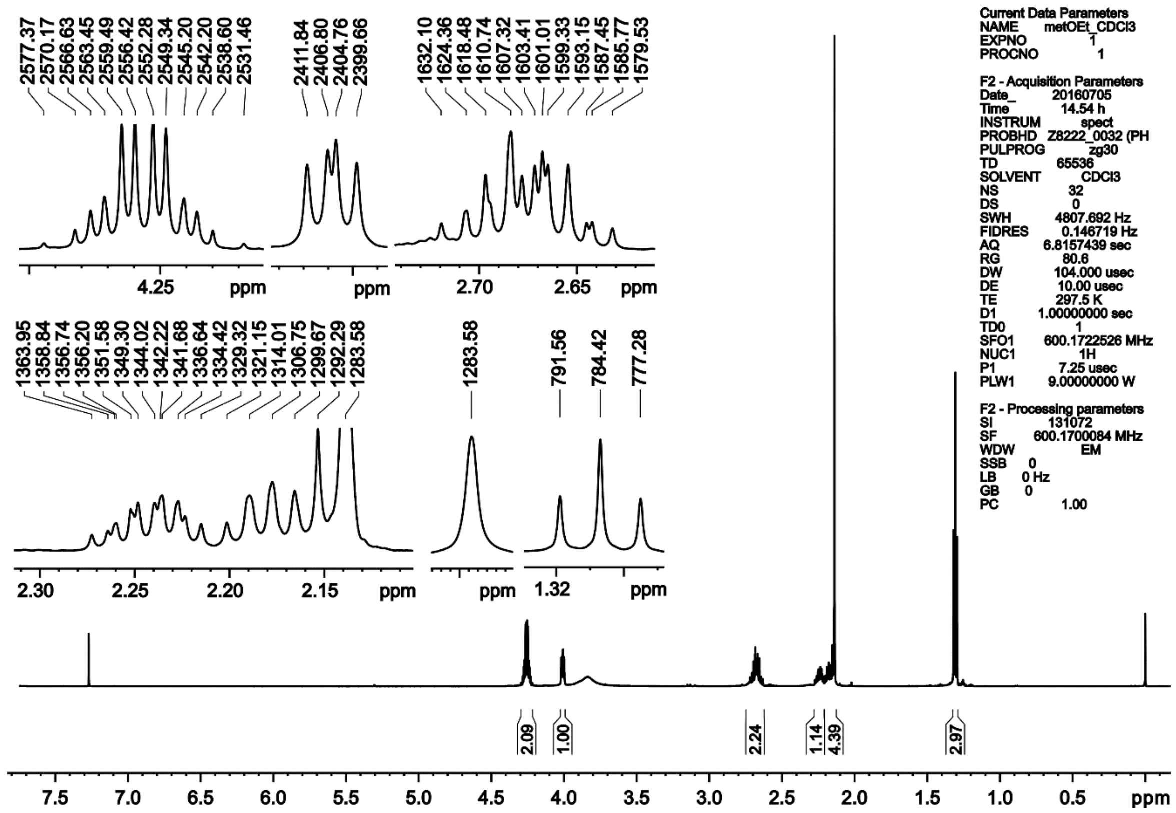

Figure S5: Compound **1** ^{1}H NMR spectrum in $CDCl_3$ at 25 °C.

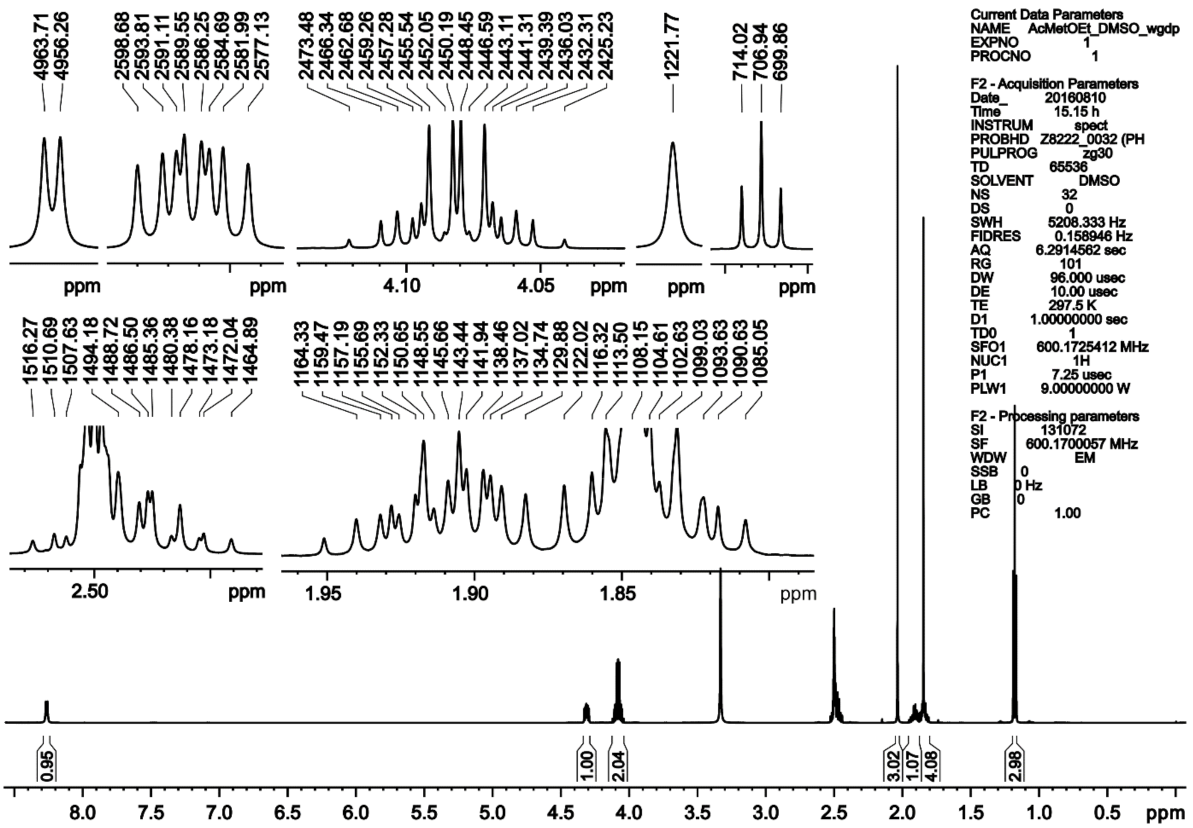

Figure S6: Compound **3** ^{1}H NMR spectrum in DMSO-d_6 at 25 °C.

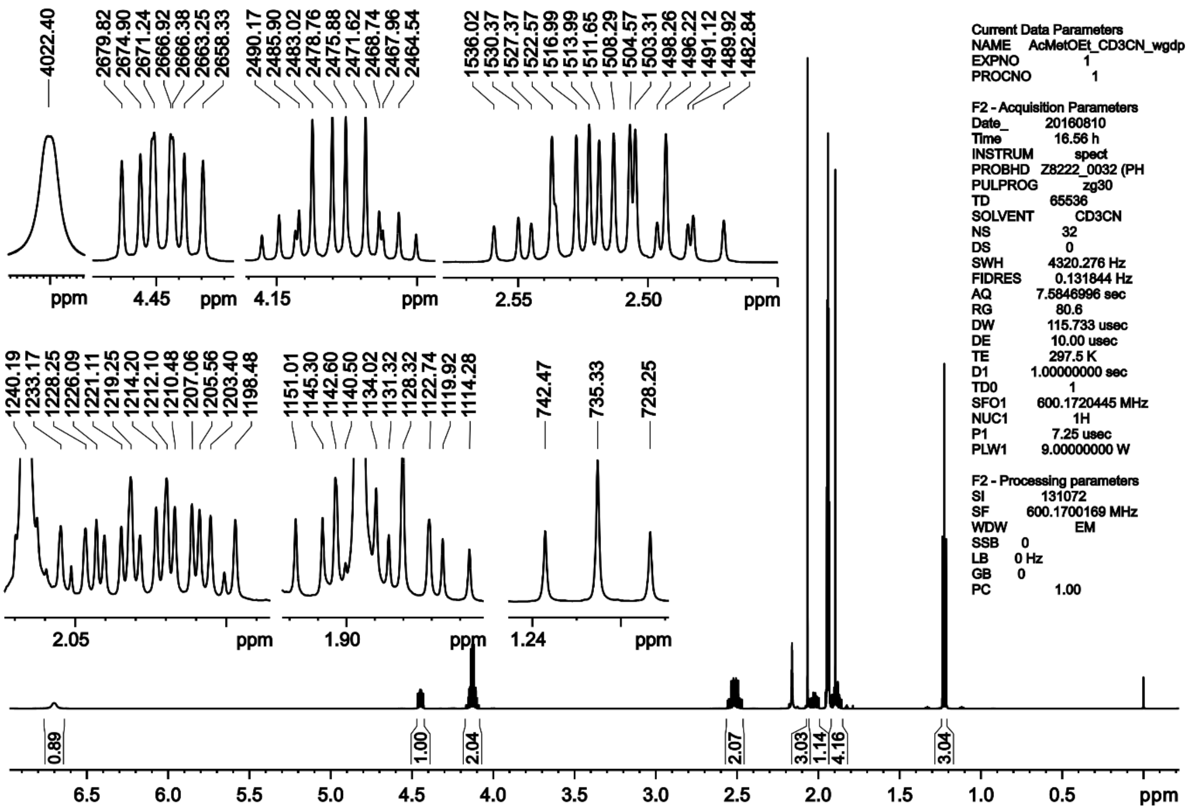

Figure S7: Compound **3** ^{1}H NMR spectrum in CD_3CN at 25 °C.

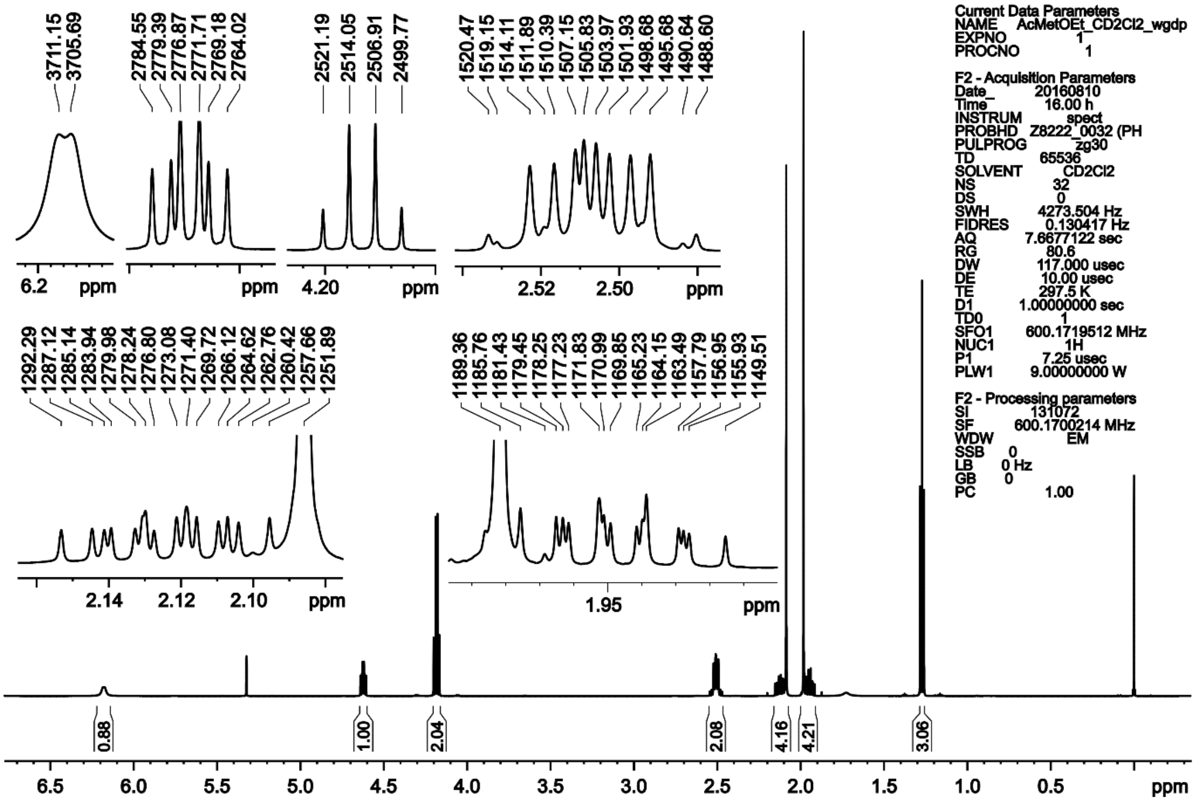

Figure S8: Compound **3** ^{1}H NMR spectrum in CD_2Cl_2 at 25 °C.

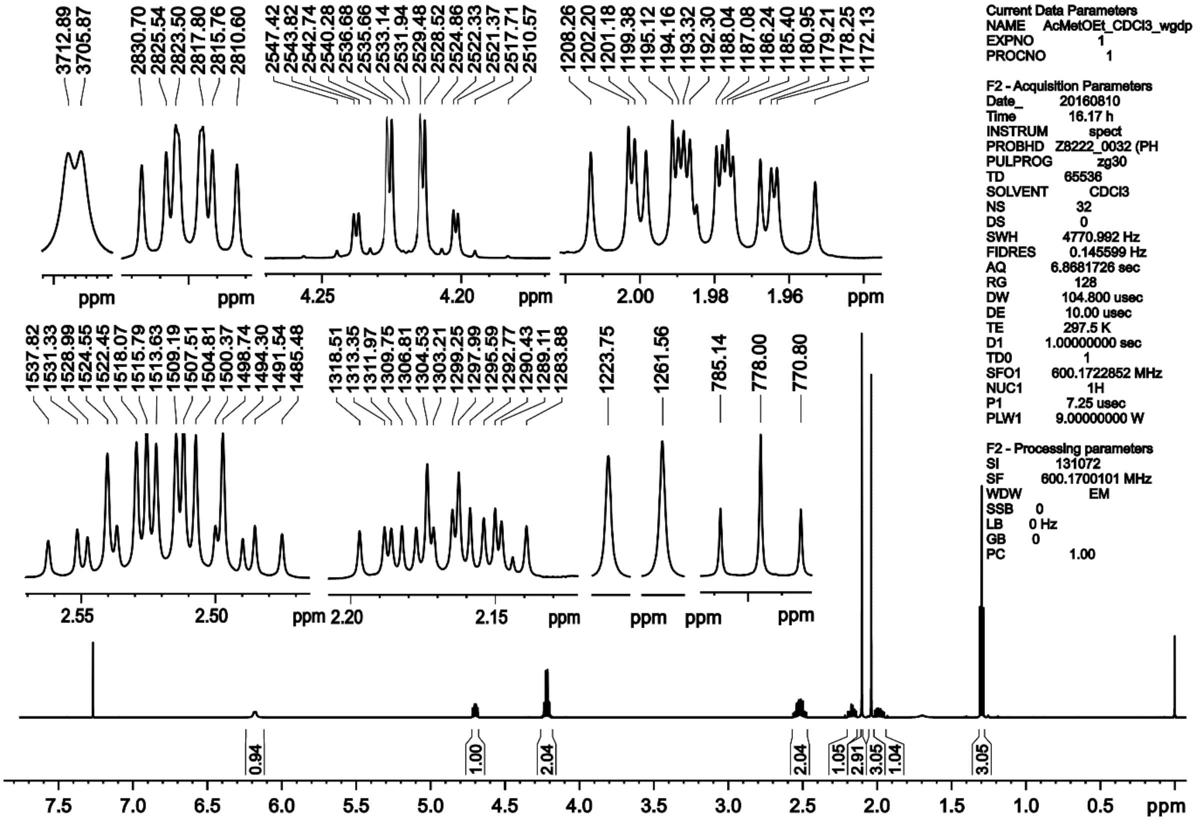

Figure S9: Compound **3** ^{1}H NMR spectrum in $CDCl_3$ at 25 °C.

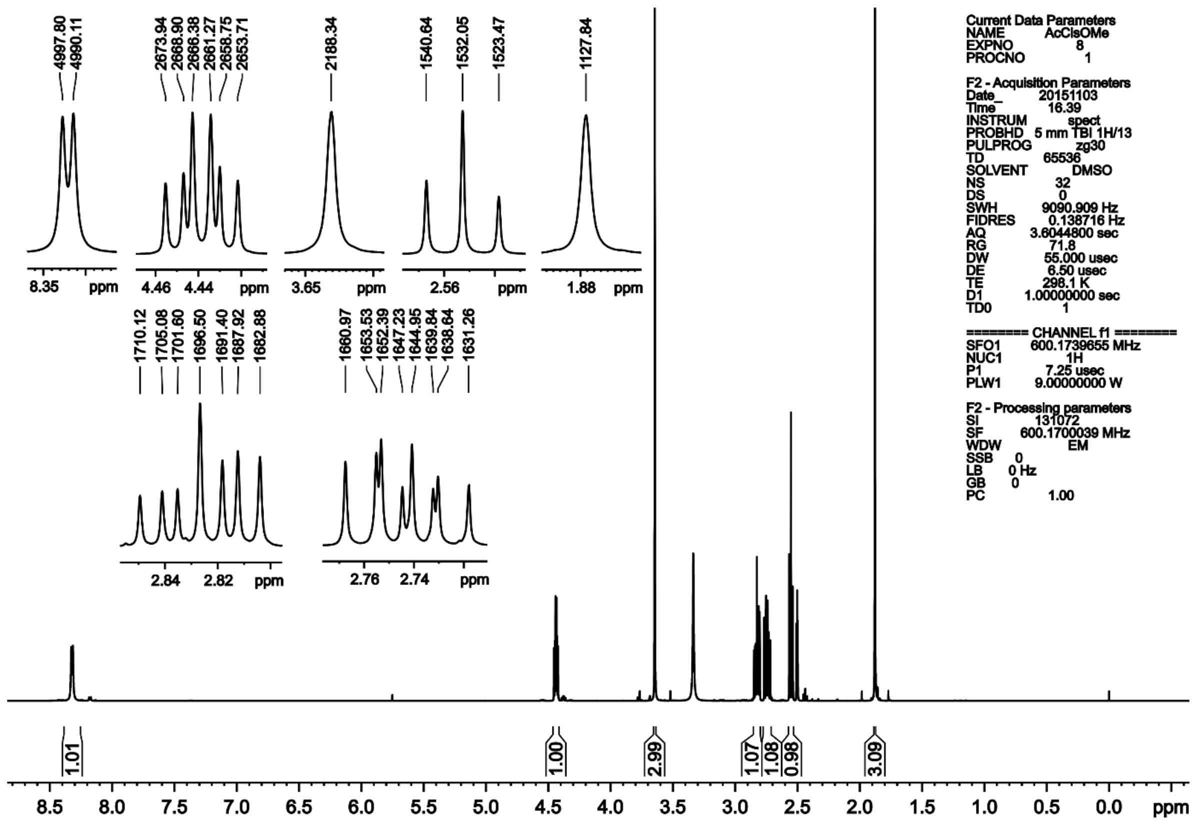

Figure S10: Compound **4** ^{1}H NMR spectrum in DMSO-d_6 at 25 °C.

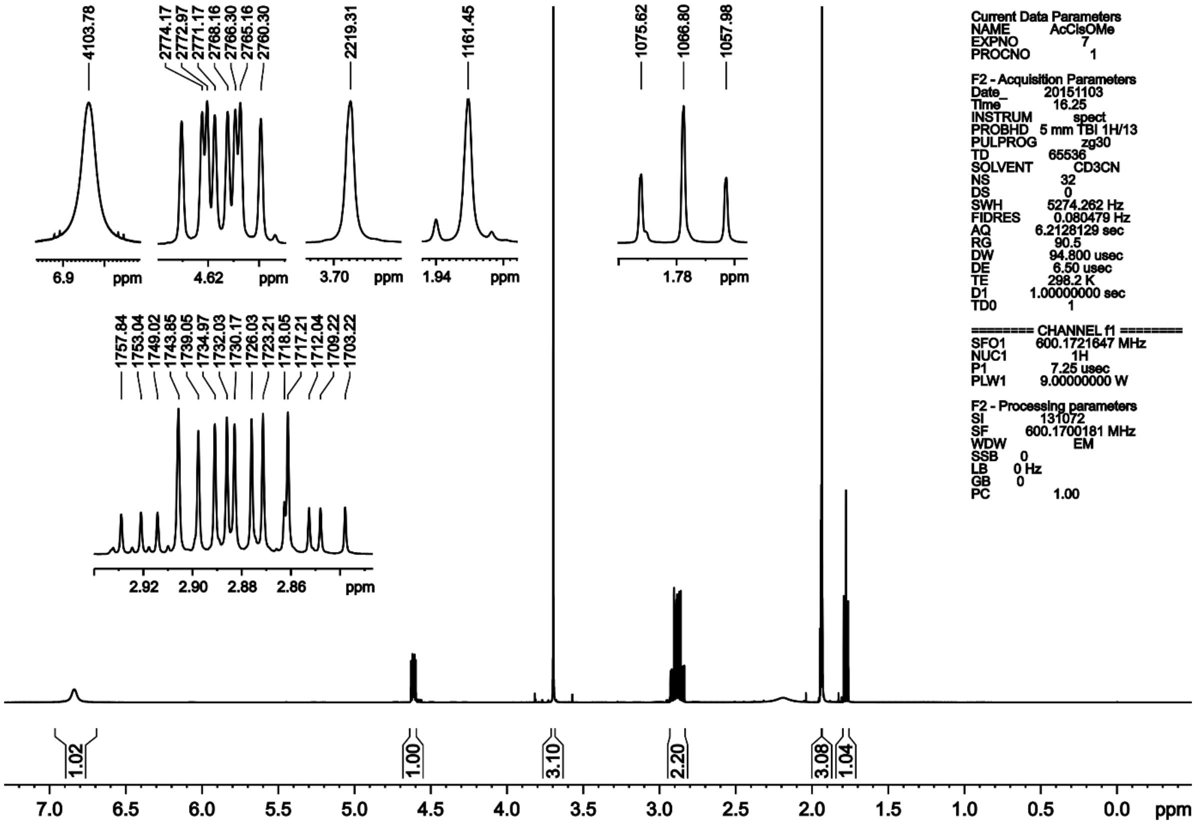

Figure S11: Compound **4** ^{1}H NMR spectrum in CD_3CN at 25 °C.

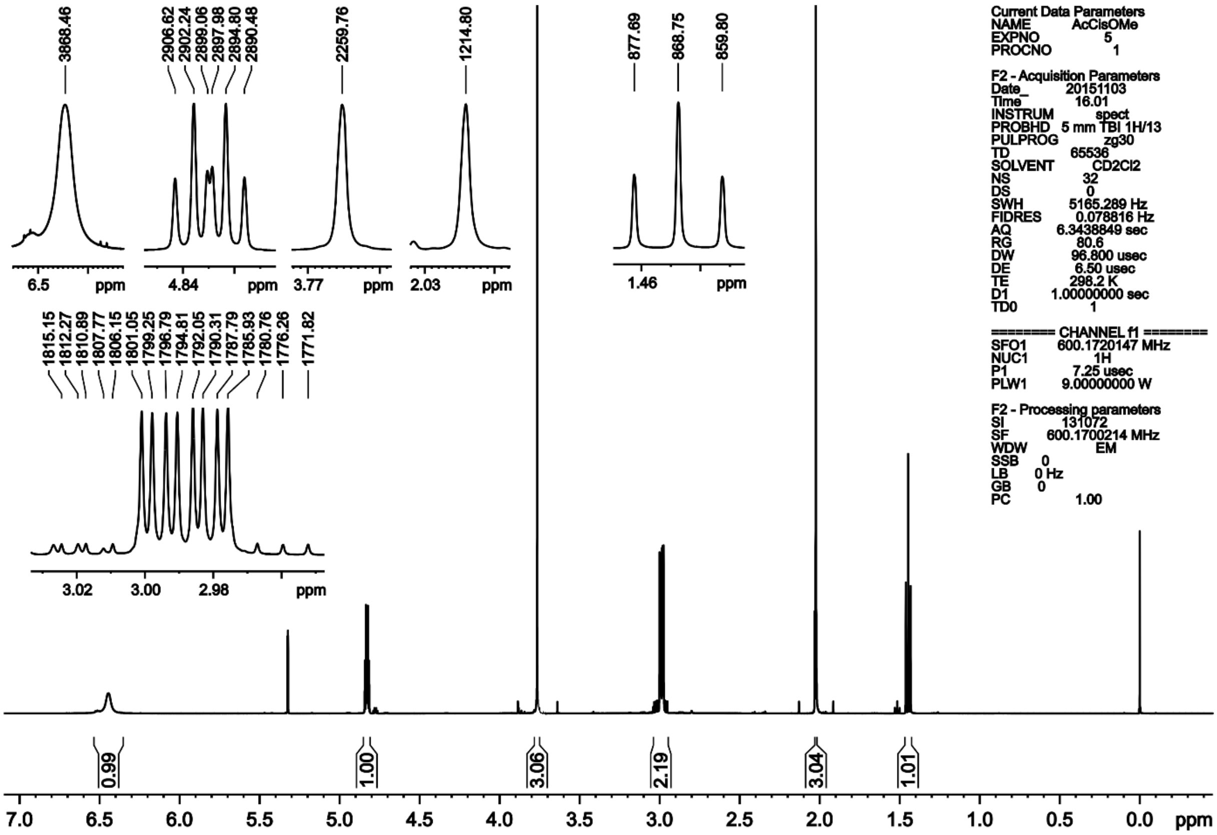

Figure S12: Compound **4** ^{1}H NMR spectrum in CD_2Cl_2 at 25 °C.

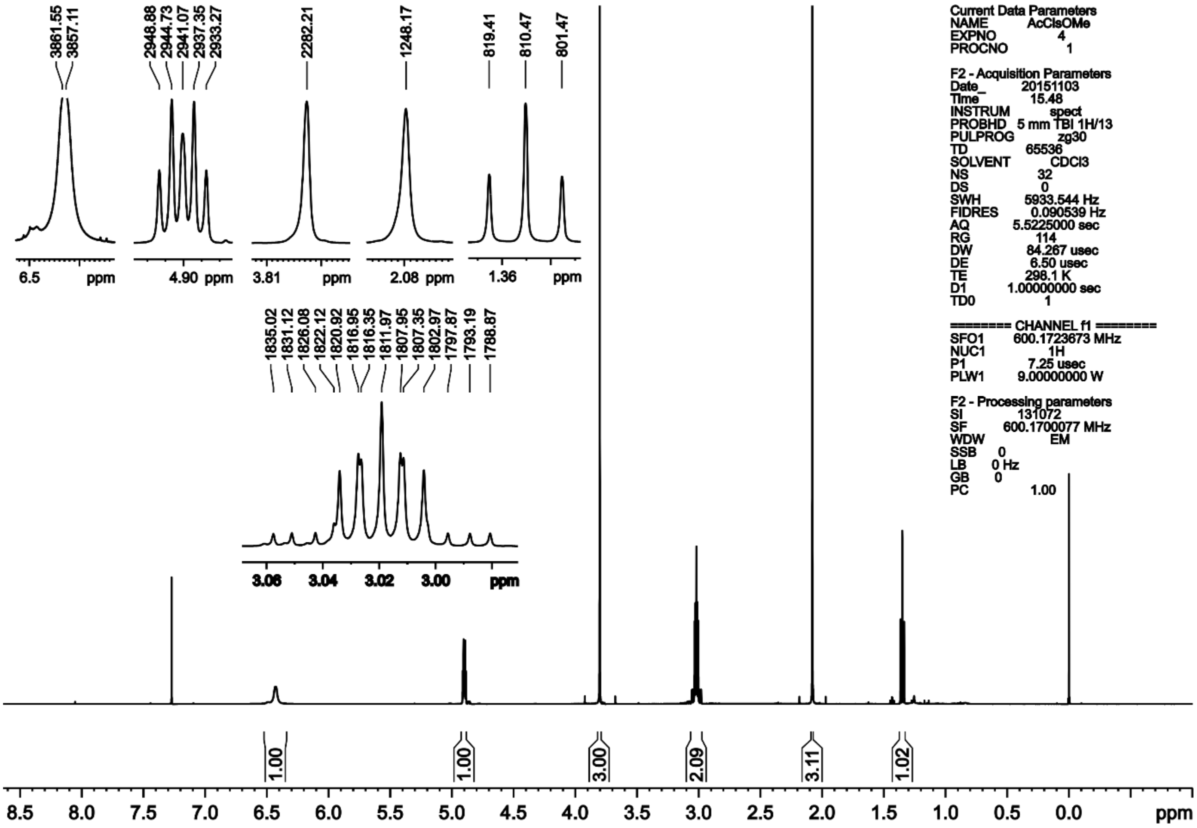

Figure S13: Compound **4** ^{1}H NMR spectrum in $CDCl_3$ at 25 °C.

CIS-I

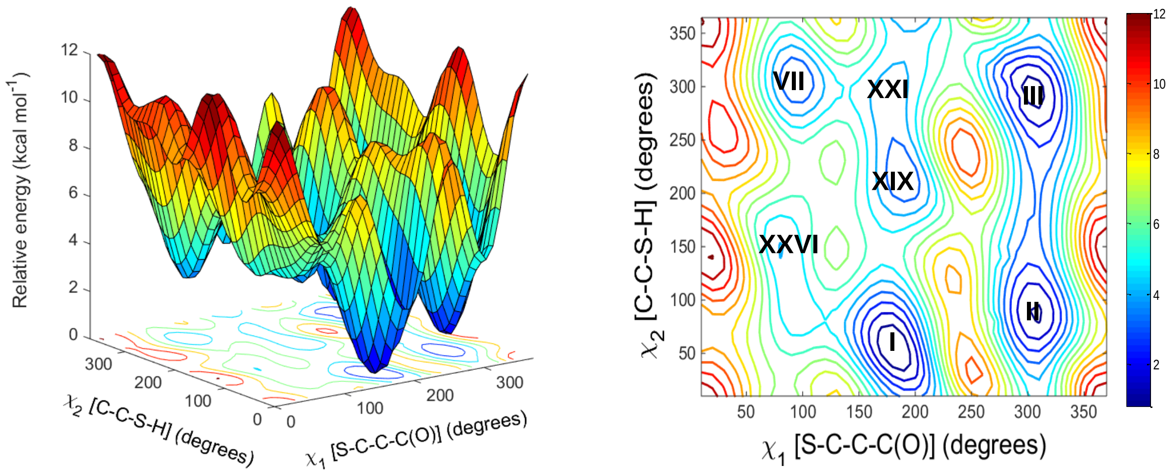

CIS-III

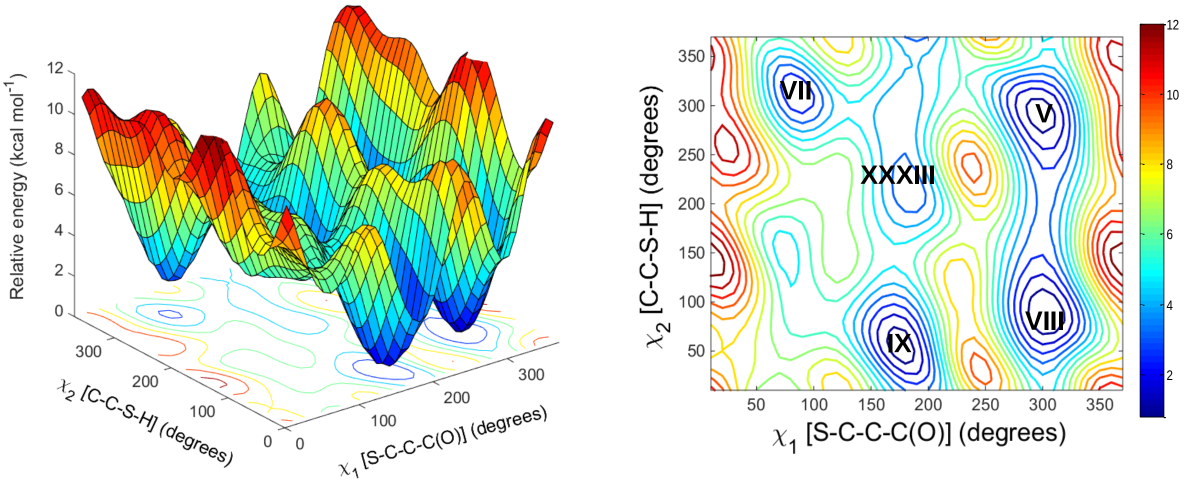

CIS-IV1

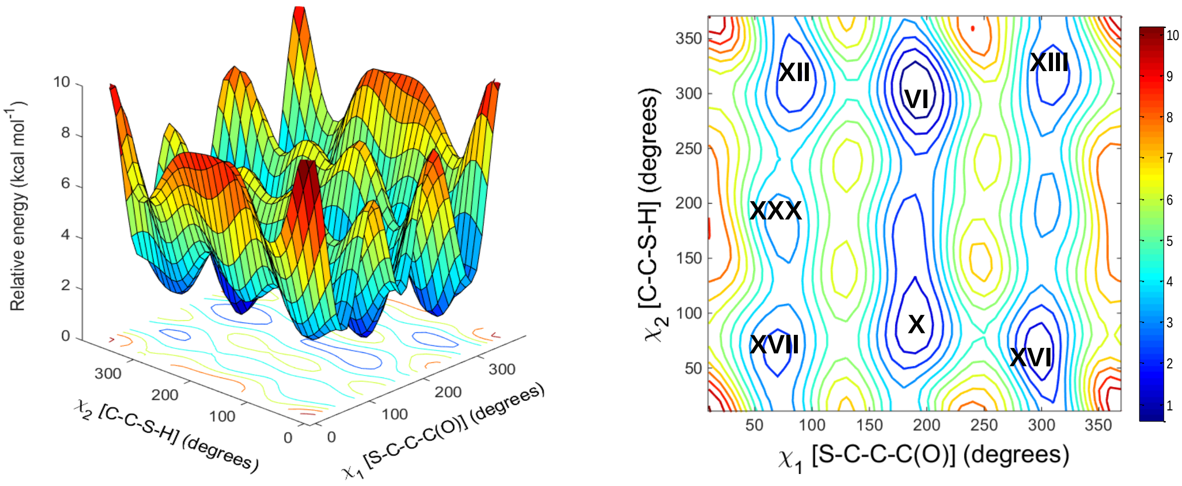

(to be continued)

CIS-IV2

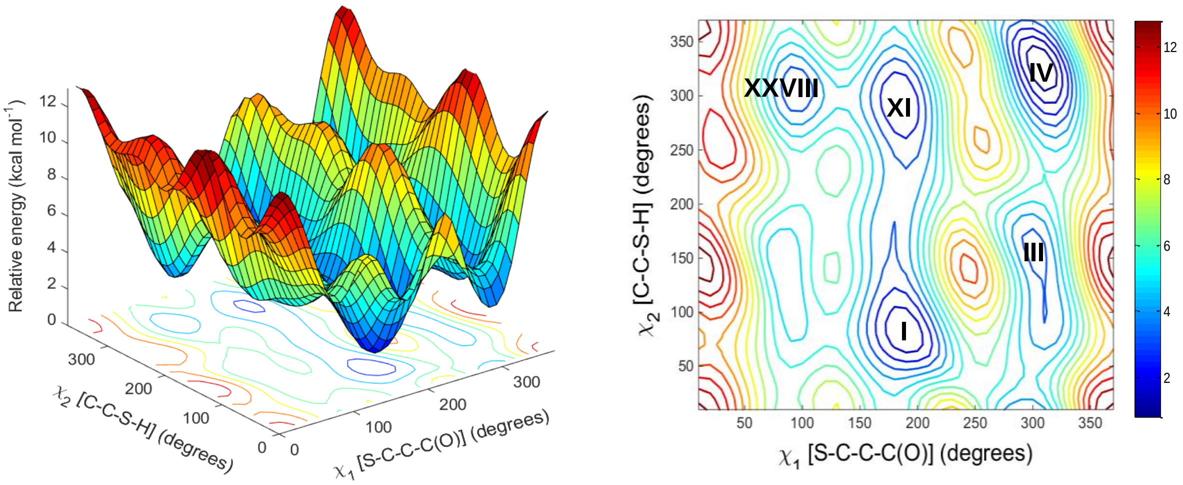

CIS-V1

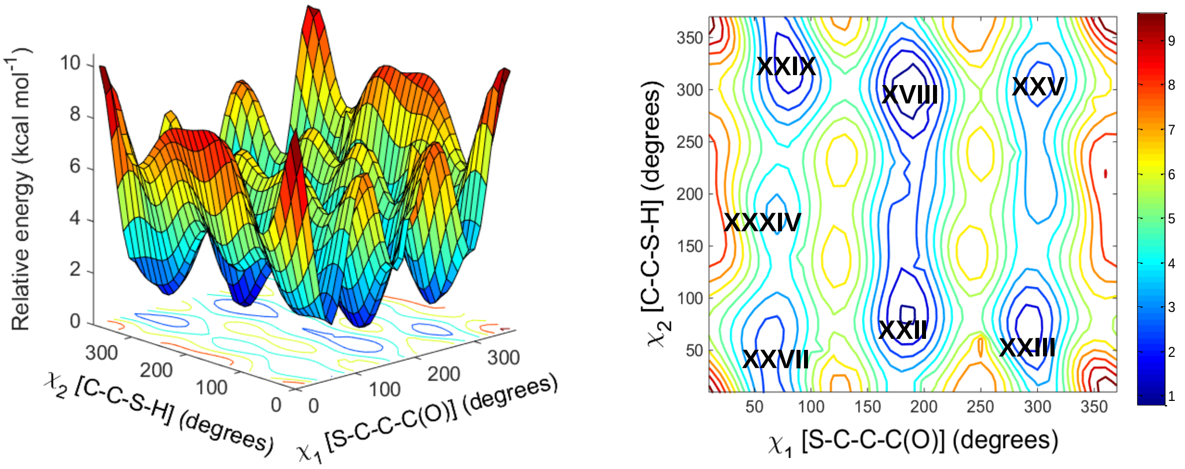

CIS-V2

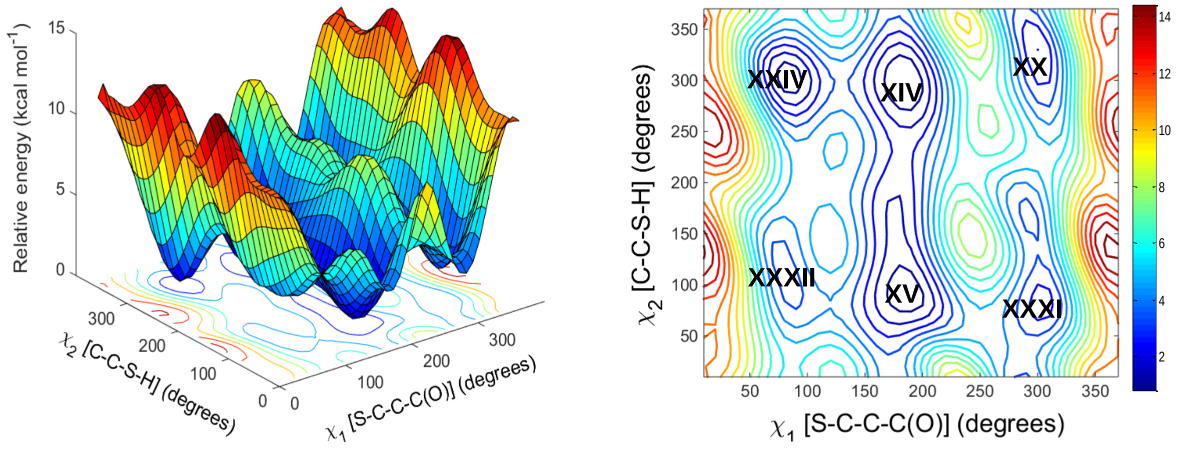

Figure S14: (a) Potential energy surfaces (PES) of the L−cysteine methyl ester **2** built by varying the χ_1 [S−C−C−C(O)] and χ_2 [C−C−S−H] dihedral angles, calculated at the B3LYP/cc−pVDZ level for the isolated molecule, and (b) their corresponding contour maps as a function of the χ_1 and χ_2 dihedral angles.

Table S1: Conformer energies[a] (E), relative energies[b] (E_{rel}), populations[c] (P), investigated dihedral angles (ω, ψ, χ_1 and χ_2)[d] and dipole moments[e] (μ) for the eight most stable conformers of the L-cysteine methyl ester **2**, obtained for the optimized geometries in isolated phase using different methods. DFT functionals used the aug-cc-pVTZ basis set. Ab initio MP2/aug-cc-pVTZ level was only the single point energy calculation from MP2/aug-cc-pVDZ optimized geometry.

conf.	Parameters	MP2	B3LYP	B3LYP-D3	CAM-B3LYP	M05-2X	M06-2X	B97-D	ωB97X-D
**Ib**	E	-760.14008	-761.42103	-761.43524	-761.25152	-761.35698	-761.23622	-761.17989	-761.28375
	E_{rel}	0.38	0.00	0.00	0.00	0.28	0.50	0.00	0.00
	E + ZPE	--	-761.28590	-761.29984	-761.11461	-761.21870	-761.09890	-761.04793	-761.14676
	E_{rel} + ZPE	--	0.00	0.01	0.00	0.26	0.68	0.00	0.00
	%P	--	36.8	22.7	31.5	15.4	9.7	29.8	29.8
	ω [O=C$_1$-O-C]	1.0	0.9	1.0	0.8	0.6	0.6	1.2	1.0
	ψ [N-C-C=O]	21.5	21.9	20.6	18.9	18.7	18.2	25.5	21.1
	χ_1 [S-C-C-C(O)]	172.8	170.8	170.9	171.1	171.3	171.5	171.5	170.9
	χ_2 [H-S-C-C]	49.0	51.9	50.8	51.8	50.6	50.8	48.3	50.5
	μ	3.05	2.55	2.52	2.53	2.55	2.44	2.62	2.56
**IIa**	E	-760.14046	-761.42007	-761.43501	-761.25083	-761.35730	-761.23687	-761.17918	-761.28350
	E_{rel}	0.14	0.61	0.14	0.43	0.08	0.09	0.45	0.16
	E + ZPE	--	-761.28491	-761.29953	-761.11385	-761.21875	-761.09998	-761.04734	-761.14629
	E_{rel} + ZPE	--	0.62	0.21	0.48	0.22	0.00	0.37	0.30
	%P	--	12.9	16.3	14.0	16.4	30.5	16.0	17.9
	ω [O=C$_1$-O-C]	1.3	0.9	1.4	0.9	1.2	1.0	1.5	1.4
	ψ [N-C-C=O]	4.2	2.2	2.8	3.7	6.1	4.9	0.3	3.5
	χ_1 [S-C-C-C(O)]	60.6	63.9	62.4	63.7	61.7	62.4	63.1	62.9
	χ_2 [H-S-C-C]	73.3	73.9	72.9	73.4	75.1	73.7	71.8	73.1
	μ	2.14	2.09	2.05	2.08	2.03	2.00	2.07	2.08

(to be continued)

**IIIa**	*E*	-760.140643	-761.42008	-761.43523	-761.25083	-761.35743	-761.23702	-761.17931	-761.28360
	E_{rel}	0.02	0.60	0.01	0.43	0.00	0.00	0.36	0.09
	E + ZPE	--	-761.28496	-761.29986	-761.11389	-761.21911	-761.09981	-761.04757	-761.14629
	E_{rel} + ZPE	--	0.59	0.00	0.45	0.00	0.11	0.22	0.30
	%P	--	13.6	23.2	14.7	23.8	25.3	20.6	17.9
	ω [O=C_1-O-C]	0.9	1.0	0.6	1.2	0.7	0.8	0.1	0.3
	ψ [N-C-C=O]	8.4	4.7	6.5	8.6	6.0	6.1	1.7	7.1
	χ_1 [S-C-C-C(O)]	61.8	66.9	63.6	65.5	62.0	62.0	64.3	63.8
	χ_2 [H-S-C-C]	73.8	74.6	71.9	74.6	72.4	73.5	68.9	72.3
	μ	2.89	2.82	2.78	2.83	2.86	2.77	2.80	2.83
**IVa**	*E*	-760.14068	-761.41990	-761.43441	-761.25091	-761.35719	-761.23658	-761.17800	-761.28321
	E_{rel}	0.00	0.71	0.52	0.38	0.15	0.28	1.19	0.34
	E + ZPE	--	-761.28474	-761.29919	-761.11393	-761.21883	-761.09948	-761.04636	-761.14595
	E_{rel} + ZPE	--	0.73	0.42	0.42	0.18	0.31	0.98	0.51
	%P	--	10.7	11.4	15.5	17.6	18.1	5.7	12.6
	ω [O=C_1-O-C]	3.5	2.4	3.1	2.6	3.9	3.8	3.2	3.3
	ψ [N-C-C=O]	9.8	8.6	8.9	9.0	8.5	9.1	8.0	9.3
	χ_1 [S-C-C-C(O)]	56.3	59.5	58.6	59.1	56.1	56.0	59.2	58.5
	χ_2 [H-S-C-C]	50.6	52.6	51.4	52.7	52.5	52.3	48.3	52.0
	μ	3.06	2.90	2.87	2.89	2.88	2.80	2.94	2.90
**Va**	*E*	-760.14001	-761.41967	-761.43473	-761.25063	-761.35703	-761.23660	-761.17845	-761.28314
	E_{rel}	0.42	0.86	0.32	0.55	0.26	0.26	0.90	0.38
	E + ZPE	--	-761.28433	-761.29904	-761.11345	-761.21854	-761.09880	-761.04640	-761.14563
	E_{rel} + ZPE	--	0.99	0.51	0.73	0.35	0.74	0.96	0.71
	%P	--	6.9	9.7	9.2	13.2	8.8	5.9	9.0
	ω [O=C_1-O-C]	0.3	0.1	0.1	0.1	0.2	0.2	0.0	0.3
	ψ [N-C-C=O]	154.3	150.2	150.0	148.6	151.0	149.7	154.9	151.6
	χ_1 [S-C-C-C(O)]	65.9	69.3	67.5	68.6	66.6	67.2	67.4	68.2
	χ_2 [H-S-C-C]	75.8	76.2	73.9	75.7	73.7	75.1	74.3	74.7

	μ	2.69	2.74	2.78	2.77	2.75	2.67	2.71	2.77

(to be continued)

(conclusion)

**VIb**	*E*	-760.13869	-761.41965	-761.43383	-761.24997	-761.35538	-761.23478	-761.17835	-761.28211
	E_{rel}	1.25	0.87	0.88	0.97	1.29	1.41	0.97	1.03
	E + ZPE	--	-761.28452	-761.29853	-761.11305	-761.21707	-761.09727	-761.04659	-761.14504
	E_{rel} + ZPE	--	0.87	0.83	0.98	1.28	1.70	0.84	1.08
	%P	--	8.5	5.7	6.0	2.7	1.7	7.2	4.8
	ω [O=C_1-O-C]	0.5	0.4	0.6	0.4	0.4	0.4	0.6	0.3
	ψ [N-C-C=O]	33.0	33.1	32.9	30.9	30.3	30.5	36.3	33.6
	χ_1 [S-C-C-C(O)]	178.8	179.7	179.2	180.0	179.7	179.2	179.6	179.5
	χ_2 [H-S-C-C]	67.7	69.6	68.2	68.9	67.1	67.8	67.8	67.7
	μ	2.61	2.51	2.48	2.56	2.59	2.49	2.43	2.52
**VIIa**	*E*	-760.13912	-761.41970	-761.43426	-761.25003	-761.35578	-761.23517	-761.17884	-761.28244
	E_{rel}	0.98	0.84	0.61	0.93	1.03	1.16	0.65	0.82
	E + ZPE	--	-761.28444	-761.29878	-761.11304	-761.21759	-761.09756	-761.04699	-761.14494
	E_{rel} + ZPE	--	0.92	0.68	0.99	0.95	1.52	0.59	1.14
	%P	--	7.8	7.4	5.9	4.8	2.4	11.0	4.3
	ω [O=C_1-O-C]	0.3	0.5	0.5	0.5	0.1	0.1	0.4	0.6
	ψ [N-C-C=O]	84.4	86.6	89.8	90.9	89.4	92.0	84.3	88.9
	χ_1 [S-C-C-C(O)]	60.8	63.7	63.0	63.2	61.7	61.9	63.2	63.0
	χ_2 [H-S-C-C]	75.2	76.7	74.1	75.9	77.0	76.1	73.8	75.5
	μ	2.82	2.80	2.88	2.90	2.86	2.81	2.79	2.89
**VIIIa**	*E*	-760.13911	-761.41888	-761.43375	-761.24962	-761.3560	-761.23549	-761.17792	-761.28225
	E_{rel}	0.98	1.35	0.93	1.19	0.90	0.96	1.23	0.94
	E + ZPE	--	-761.28350	-761.29810	-761.11245	-761.21781	-761.09795	-761.04601	-761.14481
	E_{rel} + ZPE	--	1.51	1.10	1.36	0.81	1.27	1.21	1.22
	%P	--	3.0	3.6	3.2	6.1	3.6	3.9	3.8
	ω [O=C_1-O-C]	1.6	1.0	1.4	1.0	1.6	1.4	1.6	1.5
	ψ [N-C-C=O]	176.0	175.8	176.9	173.9	174.3	174.4	179.6	178.0
	χ_1 [S-C-C-C(O)]	66.5	70.0	68.5	69.8	66.7	67.5	69.0	68.8
	χ_2 [H-S-C-C]	76.8	78.0	76.4	77.9	76.7	75.6	75.0	76.1
	μ	2.57	2.53	2.51	2.57	2.50	2.47	2.41	2.56

[a] Energies in hartree. [b] Relative energies in kcal mol^{-1}. 1 hartree = 627.5095 kcal mol^{-1}. [c] Populations in %. [d] Dihedral angles in degrees. [e] Dipole moments in debye. [f] ZPE correction included.

Experimental procedures for the synthesis of compounds 1, 3 and 4

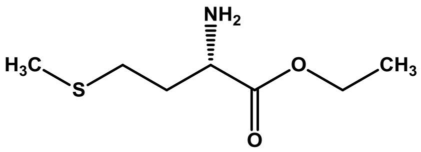

L-Methionine ethyl ester (1)[1]

Activated zinc dust (100 mg) was added to a suspension of the L-methionine ethyl ester hydrochloride (218.5 mg, 1.0 mmol) in 10 mL of dichloromethane. The reaction mixture was stirred for 3 h at room temperature. Then, the mixture was filtered, and the solvent was evaporated in vacuo. The amino acid ester free amine **1** was obtained as a colorless liquid (139.6 mg, 0.8 mmol, 77.0% yield), which was used without any further purification. **IR** (CH_2Cl_2): ν = 3317, 3234, 2985, 2922, 1728, 1574, 1435, 1298, 1227, 1084, 1019 cm^{-1}. **^{1}H NMR** (600.17 MHz, DMSO-d_6, 25 °C, TMS): δ (ppm) = 4.12 (dq, ^2J= 10.8 and ^3J=7.1 Hz, 1H), 4.10 (dq, ^2J=10.8 and ^3J=7.1 Hz, 1H), 3.55 (dd, ^3J= 7.4 and 5.6 Hz, 1H), 2.52 (m, 2H), 2.03 (s, 3H), 1.88 (m, 1H), 1.78 (m, 1H), 1.20 (t, ^3J= 7.1 Hz, 3H). **^{13}C NMR** (125.71 MHz, DMSO-d_6, 25 °C, TMS): δ (ppm) = 173.49, 61.27, 52.73, 32.65, 29.57, 14.93, 14.51. **HRMS** (TOF-ES+): *m/z* $[M+H]^+$ calculated for $C_7H_{16}NO_2S$: 178.0896; found: 178.0907.

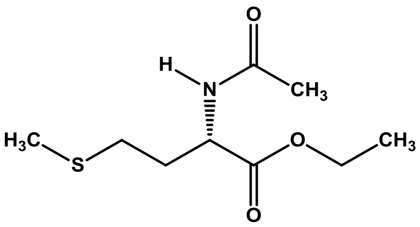

N-acetyl-L-methionine ethyl ester (3)[2]

Thionyl chloride (0.7 mL, 9.6 mmol) was carefully added to a stirred solution of *N*-acetyl-L-methionine (1.0 g, 5.2 mmol) in 10 mL of anhydrous EtOH. The reaction mixture was stirred for 3 h at 0 °C and then at room temperature for further 24 h. The solvent was removed under reduced pressure, and 5 mL of water was added to the resulting residue followed by the washing with a saturated aqueous solution of $KHCO_3$. The aqueous layer was extracted with dichloromethane (3 x 5 mL), and the combined organic layers were dried with anhydrous $MgSO_4$, filtered and concentrated

in vacuo to afford *N*-acetyl-L-methionine ethyl ester **3** (841.2 mg, 3.8 mmol, 73.4% yield) as a white solid. **IR** (KBr): ν = 3265, 2929, 2857, 1747, 1642, 1554, 1442, 1376, 1306, 1215, 1168, 1124 cm^{-1}. **^{1}H NMR** (600.17 MHz, DMSO-d_6, 25 °C, TMS): δ (ppm) = 8.26 (d, ^{3}J= 7.4 Hz, 1H), 4.31 (ddd, ^{3}J= 9.1, 7.6 and 4.9 Hz, 1H), 4.09 (dq, ^{2}J=10.8 and ^{3}J=7.1 Hz, 1H), 4.06 (dq, ^{2}J= 10.8 and ^{3}J= 7.1 Hz, 1H), 2.48 (m, 2H), 2.04 (s, 3H), 1.91 (dddd, ^{2}J= 13.8, ^{3}J= 8.7, 7.1 and 4.9 Hz, 1H), 1.84 (m, 4H), 1.18 (t, ^{3}J= 7.1 Hz, 3H). **^{13}C NMR** (150.91 MHz, DMSO-d_6, 25 °C, TMS): δ (ppm) = 172.44, 170.03, 60.95, 51.50, 31.00, 29.99, 22.72, 14.99, and 14.52. **HRMS** (TOF-ES+): *m/z* $[M + H]^+$ calculated for $C_9H_{18}NO_3S$: 220.1002; found: 220.0993.

_N_-acetyl-L-cysteine methyl ester (4) [3]

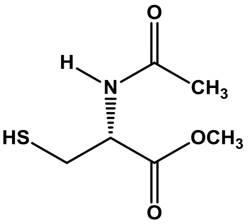

Thionyl chloride (0.5 mL, 6.8 mmol) was carefully added to a stirred solution of *N*-acetyl-L-cysteine (1.0 g, 6.1 mmol) in 10 mL of anhydrous MeOH. The reaction mixture was stirred for 1 h at 0 ºC and then at room temperature for further 2 h. The solvent was removed under reduced pressure, and 5 mL of water was added to the resulting residue followed by the washing with a saturated aqueous solution of $NaHCO_3$. The aqueous layer was extracted with ethyl acetate (3 x 5 mL), and the combined organic layers were dried with anhydrous $MgSO_4$, filtered and concentrated in vacuo to afford *N*-acetyl-L-cysteine methyl ester **4** (870.3 mg, 4.9 mmol, 80.5% yield) as a light yellow solid. **IR** (KBr): ν= 3302, 2947, 2848, 2564, 1737, 1643, 1551, 1441, 1373, 1224 cm^{-1}. **^{1}H NMR** (600.17 MHz, DMSO-d_6, 25 °C, TMS): δ (ppm) = 8.32 (d, ^{3}J = 7.5 Hz, 1H), 4.44 (td, ^{3}J = 5.1 and 7.5 Hz, 1H), 3.65 (s, 3H), 2.83 (ddd, ^{2}J = 13.7, ^{3}J = 8.5 and 5.1 Hz, 1H), 2.74 (ddd, ^{2}J = 13.7, ^{3}J = 8.6 and 7.4 and Hz, 1H), 2.55 (t, ^{3}J = 8.5 Hz, 1H), 1.88 (s, 3H). **^{13}C NMR** (150.91 MHz, DMSO-d_6, 25 °C, TMS): δ (ppm) = 171.34, 169.91, 54.96, 52.51,

25.85, 22.76. **HRMS** (TOF-EI+): *m/z* $[M + H]^+$ calculated for $C_6H_{12}NO_3S$: 178.0532; found: 178.0543.

References

1. Jullien, A.-S.; Gateau, C.; Lebrun, C.; Delangle, P. *Inorg. Chem.* **2015**, *54,* 2339–2344.

2. Bang, E.-K.; Gasparini, G.; Molinard, G.; Roux, A.; Sakai, N.; Matile, S. *J. Phys. Chem.* **1953,** *57,* 607–608.

3. Bernardes, G. J. L.; Grayson, E. J.; Thompson, S.; Chalker, J. M.; Errey, J. C.; Oualid, F. E.; Claridge, T. D. W.; Davis, B. G. *Angew. Chem. Int. Ed.* **2008**, *47*, 2244–2247.
